# Mathematical Model of Clonal Evolution Proposes a Personalised Multi-Modal Therapy for High-Risk Neuroblastoma

**DOI:** 10.3390/cancers15071986

**Published:** 2023-03-26

**Authors:** Matteo Italia, Kenneth Y. Wertheim, Sabine Taschner-Mandl, Dawn Walker, Fabio Dercole

**Affiliations:** 1 Department of Electronic, Information, and Bioengineering, Politecnico di Milano, 20133 Milano, Italy; 2 Insigneo Institute for in Silico Medicine, University of Sheffield, Sheffield S10 2TN, UK; 3 Department of Computer Science, University of Sheffield, Sheffield S10 2TN, UK; 4 Centre of Excellence for Data Science, Artificial Intelligence, and Modelling, University of Hull, Kingston upon Hull HU6 7RX, UK; 5 School of Computer Science, University of Hull, Kingston upon Hull HU6 7RX, UK; 6 St. Anna Children’s Cancer Research Institute, 1090 Vienna, Austria

**Keywords:** clonal evolution, drug delivery, evolutionary computation, mathematical model, neuroblastoma, ordinary differential equations, drug resistance, population dynamics, precision medicine

## Abstract

**Simple Summary:**

Neuroblastoma is a rare type of cancer that usually affects children. The high-risk patients’ expected survival rate is less than 50%. One reason is the lack of precision in the standard treatment protocol: a one-size-fits-all multi-modal therapy. The study presented in this paper was designed to address this deficit by optimising the use of two chemotherapeutic agents—vincristine and cyclophosphamide—during induction chemotherapy—the part of the protocol that shrinks the primary tumour before surgical removal. We combined a mathematical model and an optimisation algorithm to identify the best chemotherapy schedules for a cohort of virtual patients with different initial tumour compositions. Our results reveal novel strategies to exploit a pair of drugs with different levels of efficacy, provide a platform on which to individualise induction chemotherapy, and lay the foundation for a personalised therapy that leverages targeted therapies, multi-region sequencing, liquid biopsies, and modern computational methods to improve today’s multi-modal therapy.

**Abstract:**

Neuroblastoma is the most common extra-cranial solid tumour in children. Despite multi-modal therapy, over half of the high-risk patients will succumb. One contributing factor is the one-size-fits-all nature of multi-modal therapy. For example, during the first step (induction chemotherapy), the standard regimen (rapid COJEC) administers fixed doses of chemotherapeutic agents in eight two-week cycles. Perhaps because of differences in resistance, this standard regimen results in highly heterogeneous outcomes in different tumours. In this study, we formulated a mathematical model comprising ordinary differential equations. The equations describe the clonal evolution within a neuroblastoma tumour being treated with vincristine and cyclophosphamide, which are used in the rapid COJEC regimen, including genetically conferred and phenotypic drug resistance. The equations also describe the agents’ pharmacokinetics. We devised an optimisation algorithm to find the best chemotherapy schedules for tumours with different pre-treatment clonal compositions. The optimised chemotherapy schedules exploit the cytotoxic difference between the two drugs and intra-tumoural clonal competition to shrink the tumours as much as possible during induction chemotherapy and before surgical removal. They indicate that induction chemotherapy can be improved by finding and using personalised schedules. More broadly, we propose that the overall multi-modal therapy can be enhanced by employing targeted therapies against the mutations and oncogenic pathways enriched and activated by the chemotherapeutic agents. To translate the proposed personalised multi-modal therapy into clinical use, patient-specific model calibration and treatment optimisation are necessary. This entails a decision support system informed by emerging medical technologies such as multi-region sequencing and liquid biopsies. The results and tools presented in this paper could be the foundation of this decision support system.

## 1. Introduction

Neuroblastoma is the most common extra-cranial solid tumour in children [1]. It originates from the stem cells and progenitor cells constituting the neural crest, a transient population of multipotent cells in the vertebrate embryo [2,3,4]. Normally, these cells migrate and differentiate into diverse cell lineages, including the neurons making up the sympathetic nervous system. It is known that the amplification of the *MYCN* oncogene and activating mutations of the *ALK* oncogene can transform progenitor cells of the sympathoadrenal lineage to generate neuroblastoma [3,5]. The mutated neuroblastoma cells can interconvert between two phenotypes with different transcriptomic and epigenetic landscapes, namely a noradrenergic cell identity and a mesenchymal cell identity [6]. Their plasticity allows them to transdifferentiate into the mesenchymal state, which is more resistant to apoptosis, during chemotherapy. Within the International Neuroblastoma Risk Group (INRG) classification system [7], neuroblastoma is staged according to the patient’s age, image-defined risk factors, and tumour metastasis; while risk stratification is based on the patient’s age, stage, histology, and additional clinical and molecular parameters. Patients with very low-risk or low-risk neuroblastoma account for nearly 50% of all cases. Their event-free and overall survival rates are both above 90%. The five-year overall survival rate of the intermediate-risk cases is above 70%. By contrast, despite multi-modal therapy, over half of the patients with high-risk neuroblastoma will succumb to the disease or relapse, whereupon survival for more than three years is rare [8]. The poor prognosis for high-risk neuroblastoma is unacceptable and a contributing factor is the one-size-fits-all nature of the multi-modal therapy. This begins with induction chemotherapy to shrink the primary tumour and reduce metastases, followed by surgical removal of the tumour, consolidated by myeloablative chemotherapy with autologous haematopoietic stem cell transplantation, and finishes with maintenance therapy involving anti-GD2 monoclonal antibodies, cytokines, and a differentiating agent called isotretinoin [1]. One potential solution is to develop more precise personalised therapies to complement or even replace this one-size-fits-all therapy. The fact that different tumours have different clonal compositions is the likely reason why the one-size-fits-all multi-modal therapy does not always work. Improving our understanding of treatment resistance is an ongoing research effort, but the diverse treatment outcomes reported in the literature [8,9,10,11] indirectly prove that different tumours have different clonal compositions. Therefore, we decided to personalise chemotherapy for a virtual cohort of patients with different pre-treatment levels and patterns of drug resistance.

The development of drug resistance, both genetically conferred and acquired through phenotypic adaptation, by cancer cells is a major reason for treatment failure and the resistance mechanisms are hard to overcome. Mutations can occur throughout the entire human genome, giving the cancer cells effectively innumerable options to resist a drug [12]. Instead of attempting to disrupt the innumerable resistance mechanisms, an oncologist could take advantage of game-theoretic principles in cancer treatment. This idea has received considerable attention from the cancer research community in recent years, including a comprehensive review paper [13]. From a game-theoretic standpoint, a population of cancer cells has two exploitable weaknesses [13]. First, unlike the oncologist, it does not act rationally. Second, it can only respond to the oncologist’s first move. This decision-making problem, informed by leader-follower dynamics, is known among game theorists as a Stackelberg game. In this Stackelberg game, applying a therapeutic agent at its maximum tolerated dose (MTD) until disease progression is clinically observable is suboptimal, because this strategy fails to exploit three vulnerabilities in the cell population.

1 The clonal competition between the treatment-sensitive, treatment-resistant, and non-malignant cell populations weakens the total cell population. For example, an evolution-guided application of paclitaxel was found to keep resistant cancer cells in check, thus prolonging the progression-free survival in preclinical breast cancer models [14].
2 If the oncologist could hypothetically predict which mutations will be selected by the therapeutic agents, an evolutionary trap could theoretically be created (called a sucker’s gambit in the review [12]). In fact, the treatment-sensitive population could theoretically be maintained indefinitely by cycling between two complementary agents (evolutionary herding). It is known from experimental data that targetable mutations and alterations of oncogenic pathways in neuroblastoma are selected by chemotherapeutic agents and enriched at relapse [15]; examples are de novo mutations in *ALK* [16] and the genes encoding the RAS-MAPK pathway [17]. In fact, drugs targeting specific molecular aberrations in neuroblastoma are under active development and ALK inhibitors are the most notable examples because they are frontline treatment options [18,19]. Although neuroblastoma can develop resistance to ALK inhibitors too, the resistance mechanisms involved create other vulnerabilities, such as hypersensitivity to MEK inhibition [20].
3 Chemotherapy will break the total cell population into smaller, fragmented (spatially distinct) [21], and genetically homogeneous (discussed above) cell populations. They are potentially vulnerable to even tiny stochastic perturbations induced by drugs or hypoxia. For instance, in a tumour, cells must cooperate to generate an angiogenic signal [22], so targeted therapies would kill them most effectively after the tumour breaks into clusters and before they can reconnect to build new blood vessels. To exploit the unique vulnerabilities of small populations, it is necessary to switch therapeutic agents when the vulnerabilities emerge.

In line with the idea of exploiting leader–follower dynamics, this paper proposes to improve the existing one-size-fits-all multi-modal therapy for high-risk neuroblastoma by incorporating evolutionary principles in a manner specific to each tumour’s unique pre-treatment clonal composition. This patient-specific strategy creates small, fragmented, and genetically homogeneous cell populations by induction chemotherapy before employing targeted therapies against the enriched mutations. Its implementation entails predicting, for each individual patient, the optimal schedule of drug administration during induction chemotherapy, as well as when to switch to the second strike with targeted therapies, which can potentially form an extra treatment block before consolidation chemotherapy. This, in turn, entails estimating the patient’s tumour composition—number of clones, their sizes, and their degrees of resistance—from personalised biomarkers such as the mutation and expression profiles of the tumour.

Following a review of the chemotherapeutic agents used to treat neuroblastoma, as well as the in vitro and in vivo data available for model calibration, we developed a mathematical model of neuroblastoma clonal evolution in the presence of vincristine (VCR or O) and cyclophosphamide (CPM or C), two complementary agents adopted in the rapid COJEC chemotherapy regimen [9,10]. This minimalist approach was adopted because the intention was to illustrate how our proposal works in principle. Then, we calibrated the model. Finally, we used a custom algorithm combining a genetic algorithm with a local search method to find the optimal drug schedules for 36 tumour compositions. In the next section, we will provide a quick guide to the model structure, calibration pipeline, and optimisation method. More technical details about the methodology, including calibration and validation, can be found in the Supplementary Materials file, which also includes a mini-review of the chemotherapeutic drugs currently used to treat neuroblastoma. After the quick guide, we will present the optimisation results. We will conclude with a discussion of the general principles inferred from these results and of our work in relation to the literature. One key point is the potential of our model and optimisation approach to absorb new experimental results and reveal further exploitable evolutionary dynamics. Another is their ability to utilise patient-specific data to establish a decision support system to personalise the one-size-fits-all multi-modal therapy which is the standard today.

## 2. Quick Guide to Methodology

**Figure 1:**
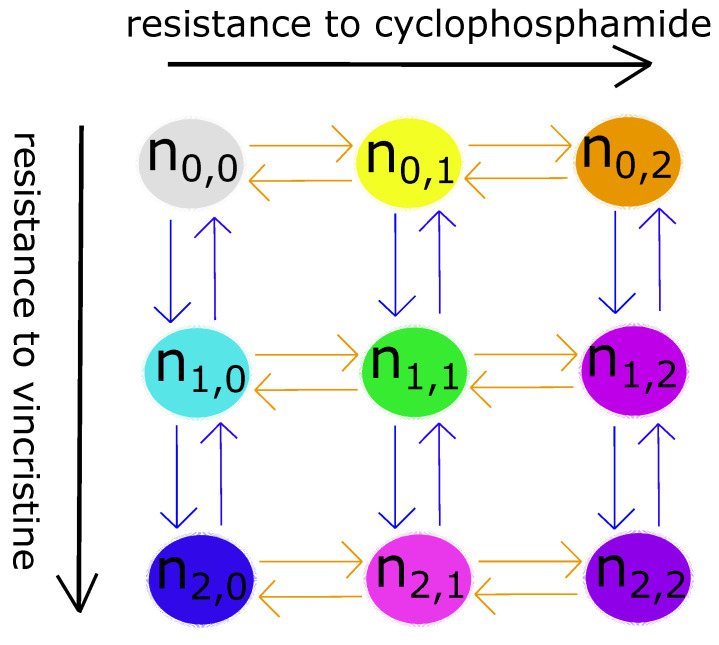
The mathematical model’s population structure. Each circle represents a subpopulation (clone) with two drug-specific resistance levels. Each $n_{i,j}$ is the size of a clone (cell count). Each arrow indicates a possible mutation step.

**Mathematical model of clonal evolution and pharmacokinetics:** Our model describes the average dynamics of a structured population of cells, divided into nine different clones (genotypes). A key model assumption is that a cell undergoes only three processes: growth (balance between division and natural death), mutation, and drug-induced death. The rates of these processes depend on the cell’s genotype, phenotype, and environment. It assumes that a cancer cell (neuroblastoma cell) can have three levels of genetically conferred resistance to a chemotherapeutic agent: none, mild, and strong. For the sake of simplicity, the model considers two drugs only, resulting in nine clones whose resistance levels with respect to the drugs are denoted by *i* and *j*, respectively; where *i* and *j* can take the values of 0, 1, and 2. Although Figure 1 is relevant to the particular case where *i* denotes the level of resistance to vincristine (VCR) and *j* denotes the level of resistance to cyclophosphamide (CPM), it also describes the mathematical model’s general population structure. Furthermore, it assumes that regardless of its genotype (the clone it belongs to), the cell can phenotypically adapt (plasticity) to prolonged exposure to the drugs by altering its gene expression pattern, including epigenetic alterations [23]. For example, cancer cells and neuroblastoma cells in particular can upregulate their DNA repair proteins and drug efflux pumps to acquire multi-drug resistance [24]. As these alterations cost energy in the form of ATP, the model assumes phenotypic adaptation is at the expense of biomass production and growth [25]. Although it is known from experiments that chemotherapy enriches targetable mutations and activates oncogenic pathways [15], the model does not include this phenomenon, which we decided to be beyond the scope of this pilot study.

The main model equation describes the evolution of a genetically identical subpopulation—a clone—of neuroblastoma cells in terms of the three basic processes, as follows

(1) $$\frac{dn_{i,j}\left(t\right)}{dt}=\frac{G(t)}{1+\phi_{1}\left(\tau \right)}-\frac{M(t)}{1+\phi_{1}\left(\tau \right)}-\frac{D(t)}{1+\phi_{2}\left(\tau \right)},$$

where $n_{i,j}$ is the number of cells in the clone, *G* is the logistic growth rate of the clone in the presence of competing clones ($n_{k,l}$, which includes $n_{i,j}$), *M* is the difference between its rate of mutation into other clones and the rate of other clones mutating into it, and *D* is the rate of its drug-induced death. In this model, each clone can only mutate into a clone whose resistance level is one above or below its own, such as from ‘none’ to ‘mild’ or from ‘strong’ to ‘mild’ (arrows in Figure 1). All three rates have the same units (h${}^{-1}$) and neglect phenotypic adaptation to chemotherapy. Phenotypic adaptation is modelled by the dimensionless functions $\phi_{1}$ and $\phi_{2}$. These functions increase with time as long as the tumour remains exposed to at least one of the two drugs. They reduce all three terms in the manner detailed in the Supplementary Materials file.

The functional form of *G* is $\left(1-\frac{\sum_{k,l}n_{k,l}}{K}\right)\left(r_{i,j}n_{i,j}\right)$, where *K* is the maximum number of cells the tumour can carry and $r_{i,j}$ is the clone’s growth rate (h${}^{-1}$). *M* is $\mu \left(1-\frac{\sum_{k,l}n_{k,l}}{K}\right)\left(\gamma_{i,j}r_{i,j}n_{i,j}-\sum_{p,q}r_{p,q}n_{p,q}\right)$, where μ is the dimensionless mutation rate, $\gamma_{i,j}$ counts the genotypes the clone associated with $n_{i,j}$ can mutate to, and the subscripts *p* and *q* denote these eligible clones. *D* is $\sum_{d}m_{d}^{i,j}\left(c_{d}\right)n_{i,j}$, where $c_{d}$ is the concentration of drug *d* (mass *per* unit of body surface: mg m${}^{-2}$ for VCR and g m${}^{-2}$ for CPM) and $m_{d}^{i,j}$ is the $n_{i,j}$ clone’s concentration-dependent mortality rate (h${}^{-1}$) associated with drug *d*. The model uses the functional form $m_{d}^{i,j}\left(c_{d}\right)=\frac{m_{d,0}^{i,j}c_{d}}{1+\alpha_{d}c_{d}^{\beta_{d}}}$, where $0<\beta_{d}\leq 1$, to describe drug-induced mortality rate. It reflects how the rate’s response to an increase in drug concentration saturates when the concentration is high. If $\beta_{d}=1$, the limiting rate is $m_{d,0}/\alpha_{d}$. For the details on the $m_{d}^{i,j}\left(c_{d}\right)$ function, the reader is referred to the Supplementary Materials file.

For the sake of simplicity, drug delivery is assumed to follow first-order pharmacokinetics in the model, resulting in the following equation governing the concentration dynamics of the two drugs (d=1 and d=2),

(2) $$\;\frac{dc_{d}\left(t\right)}{dt}=\omega_{d}-z_{d}c_{d}\left(t\right),$$

where $\omega_{d}$ and $z_{d}$ are the delivery rate (mg m${}^{-2}$ h${}^{-1}$ for VCR and g m${}^{-2}$ h${}^{-1}$ for CPM) and degradation rate (h${}^{-1}$) of drug *d*, respectively. The two delivery rates ($\omega_{d}$) are the control variables that can be manipulated in a clinical setting.

In addition to the biological and pharmacokinetic assumptions already stated, the model assumes that the spatial variations in the tumour can be neglected. Another key assumption is that dealing with large numbers of cells and molecules makes any stochastic events negligible, thus justifying the use of continuous variables and deterministic equations.

Overall, there are nine equations based on Equation (1) and two equations based on Equation (2). The time-dependent continuous state variables are $n_{i,j}$ (nine) and $c_{d}$ (two), while the control variables are $\omega_{d}$ (two). The Supplementary Materials file contains a more in-depth description of the model and its underlying assumptions.

**Summary of model assumptions.** For the sake of clarity and convenience, we summarise the key model assumptions here.

1 A population of neuroblastoma cells (a clone) can undergo three processes only: growth (division minus natural death), mutation, and drug-induced mortality.
2 Each clone follows logistic growth, limited by the other clones and the total carrying capacity (clonal competition).
3 A neuroblastoma cell has three levels of genetic resistance to a drug: none, mild, and strong. It can only mutate and enter a clone whose resistance level is directly above or below its own. Mutation occurs randomly—uniformly in all directions—in the absence of drugs (selective pressures). Therefore, the mutation term is simply the growth term multiplied by the mutation rate.
4 In addition to genetically conferred resistance, a neuroblastoma cell can phenotypically adapt to drugs after prolonged exposure to them. Adaptation costs energy, so both cell death and growth will decrease as a result. The extent of decrease depends linearly on the length of the exposure period.
5 Drug delivery follows first-order pharmacokinetics.
6 Spatial variations and stochastic effects are both assumed to be negligible.

**Aggregation of experimental data for model calibration and validation:** We carried out a review of the chemotherapeutic agents currently used to treat neuroblastoma. Based on our review (see the Supplementary Materials file), vincristine (d=1) and cyclophosphamide (d=2) affect the M-phase and the entirety of the cell cycle, respectively, so their mechanisms of action are complementary. We also found sufficient data about them for model calibration. Therefore, these drugs were chosen for inclusion in the model. The resistance levels with respect to vincristine and cyclophosphamide are denoted by *i* and *j*, respectively. In order to inform the model, experimental data regarding neuroblastoma cells’ responses to vincristine and cyclophosphamide were aggregated from different sources [26,27,28,29,30,31,32,33]. First, armed with in vitro data about the growth kinetics of neuroblastoma cell lines with different levels of drug resistance, collected in the absence of treatment, we calibrated the growth rates ($r_{i,j}$). Second, we calibrated the drug-induced death rates, specifically the $m_{d}^{i,j}\left(c_{d}\right)$ function for both drugs. This step was informed by in vitro experiments involving differentially resistant cell lines in media with different drug concentrations (deducing the types and magnitude of drug dose-dependences on sensitive neuroblastoma cells, and re-calibrating the magnitude only on resistant neuroblastoma cells), as well as a different set of in vivo experiments performed on mice because compatible in vitro data were not available. Using the data, we deduced how the mortality rates of sensitive neuroblastoma cells and drug doses are related, qualitatively and quantitatively. Then, we adjusted the quantitative aspects of this relation for resistant neuroblastoma cells.. Third, we parameterised the functions modelling phenotypic adaptation—$\phi_{1}$ and $\phi_{2}$—based on in vitro data. The remaining parameters—*K*, μ and $z_{d}$—were taken from the literature directly or indirectly after adjusting them for this model. Finally, after fixing every parametric value, we used the calibrated model to replicate experimentally observed trends with success, thus validating both the model design and calibration. The calibrated parameters indicate that having genetically conferred resistance to a drug comes at the expense of a lower growth rate in the absence of the drug. The Supplementary Materials file includes a more technical discussion of our calibration pipeline, including its limitations. Table 1 shows the calibration results summarising the model parameters, including their symbols, numerical values, units, and physical meanings.

**Optimising drug schedules using a genetic algorithm and gradient descent.** The drug doses and timings of administration, as well as the overall duration of induction chemotherapy, constitute the strategies in the Stackelberg game described in the introduction. Mathematically, this strategy is encoded by the two $\omega_{d}$ variables in Equation (2). As indicated in the introduction, it is currently difficult or impossible to infer a tumour’s clonal composition before treatment. Therefore, we decided to evaluate a broad range of resistance levels: zero, five, 10, 15, 20, and 25% of the total cell count before treatment, which was set to be half of the carrying capacity in all cases ($K/2=5\cdot 10^{9}$ cells, corresponding to 5 cm${}^{3}$). In each case, we found the optimal chemotherapy schedule by minimising the tumour size (sum of the nine $n_{i,j}$ values) at the end of the schedule, i.e., after two weeks from the beginning of the last cycle. Although the tumour’s final composition is arguably as important as its final size, we did not include it in our objective function because the induction chemotherapy stage is followed by other modes of treatment, such as surgery, immunotherapy, and radiotherapy, which do not necessarily depend on the tumour’s final composition in terms of the resistance to chemotherapy. The fundamental unit of a two-week cycle was adopted, so only the number of cycles and the two doses in each cycle were varied and optimised. We decided to limit our study to 12 or fewer cycles due to concerns about patient toxicity. In each cycle of a simulation, up to 2 mg m${}^{-2}$ of VCR and 2 g m${}^{-2}$ of CPM—their MTDs—were administered in the first 48 and 56 h, respectively [34,35]. In a clinical setting, VCR is administered intravenously as a solution, while CPM is administered as powder [34,35], hence the different periods of administration in our studies. We found literature values for the clearance rates in the two pharmacokinetic equations [36,37,38,39]. To be consistent with these studies, optimal drug schedules for tumours with different clonal compositions were obtained for a three-year-old child, 80 cm in height and 15 kg in weight, just like the child in whom the chosen clearance rates were measured. The use of a genetic algorithm allowed us to search multiple, disconnected regions of the solution space, while a method based on the gradient descent was used to search the vicinity of the solution proposed by the genetic algorithm more thoroughly. For the technical details pertaining to our optimisation algorithm, please consult the Supplementary Materials file.

## 3. Results

This section presents our optimisation results in three groups. The first group includes the results concerning tumours comprised of VCR-resistant and fully sensitive cells before treatment. The second group is pertinent to tumours comprised of CPM-resistant and fully sensitive cells before treatment. The third group concerns tumours comprised of VCR-resistant, CPM-resistant, and fully sensitive cells before treatment. As explained in the introduction, the clonal composition of a patient is currently unknown before treatment. Thus, for each combination of resistant clones, we considered five initial levels of resistance: five, 10, 15, 20, and 25 % of the total cell count, which was set to be half of the carrying capacity ($K/2=5\cdot 10^{9}$ cells).

In each optimisation problem, the initial clonal composition was fixed before optimising both the number of cycles and the doses in each cycle. In this manuscript, an optimal schedule is one that can minimise the final size (the size after two weeks from the beginning of the last cycle) of a tumour with a particular clonal composition before treatment. The Supplementary Materials file contains a comprehensive description of the set up and solution process of the optimisation problems.

The one-size-fits-all protocol used in the induction phase of the multi-modal therapy is called rapid COJEC [9,10], which uses fixed doses of chemotherapeutic agents in eight two-week cycles. We solved an optimisation problem concerning a fully sensitive population of cancer cells and found that using VCR and CPM at their MTDs for eight cycles—similar to rapid COJEC—minimised the final tumour size. Specifically, almost one percent of the initial population remained at the end of the simulated treatment, corresponding to a clinically complete response according to the International Neuroblastoma Response Criteria (INRS) [40]. The criteria include multiple metrics in addition to the final tumour size. However, considering the final size only, a complete response (CR) refers to a size reduction above 95%, a partial response (PR) refers to a size reduction above 40%, a progress disease (PD) occurs when the size increases by more than 20%, and a stable disease (SD) occurs when the size does not change enough to qualify as either PR or PD. Moreover, during the simulated treatment with MTDs, most of the neuroblastoma cells remained sensitive. At the end of the simulation, 94.61% of them were still sensitive. That our solution agrees with the standard clinical practice, which does not take resistance into account, supports the validity of our model. In this manuscript, this is referred to as the default schedule. The three groups of results in this section are answers to two questions concerning resistant tumours. Can we improve the default schedule by changing the number of cycles? What are the optimal doses in each cycle?

### 3.1. Mixtures of Fully Sensitive and VCR-Resistant Cells

**Figure 2:**
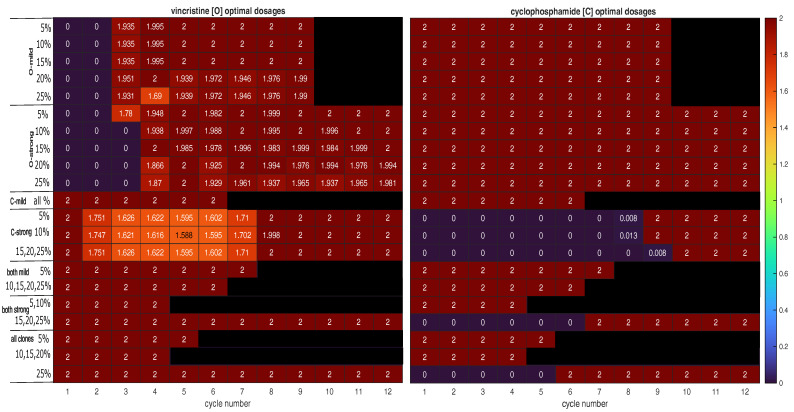
Optimal chemotherapy schedules for tumours with different initial clonal compositions. The first block is pertinent to mixtures of mildly VCR-resistant and fully sensitive cells; the second block, mixtures of strongly VCR-resistant and fully sensitive cells; the third block, mixtures of mildly CPM-resistant and fully sensitive cells; the fourth block, mixtures of strongly CPM-resistant and fully sensitive cells; the fifth block, mixtures of mildly VCR-resistant, mildly CPM-resistant, and fully sensitive cells; the sixth block, mixtures of strongly VCR-resistant, strongly CPM-resistant, and fully sensitive cells; and the last block, mixtures of VCR-resistant (mildly and strongly), CPM-resistant (mildly and strongly), and fully sensitive cells. VCR and O both denote vincristine, while CPM and C both denote cyclophosphamide. The VCR doses are in mg m${}^{-2}$ and the CPM doses are in g m${}^{-2}$. The percentages quantify the extent to which a virtual tumour was initially occupied by resistant cells. The Supplementary Materials file contains enlarged copies of these two heat maps.

**Figure 3:**
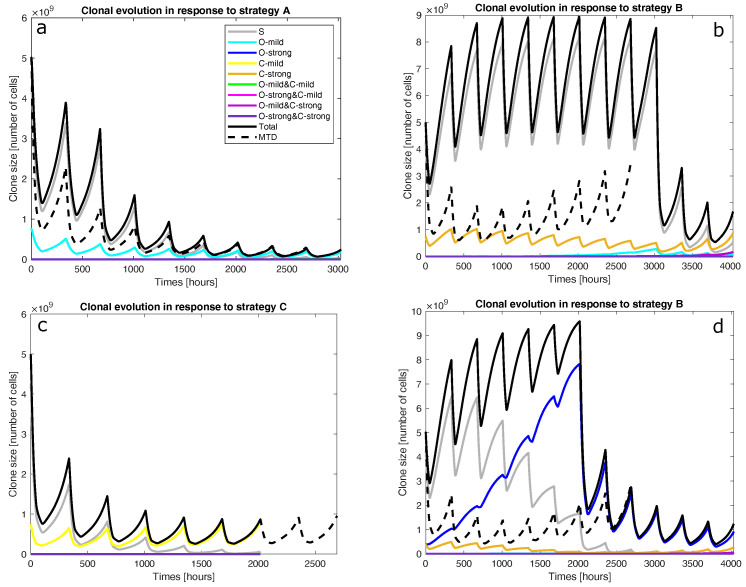
Population dynamics of the nine clones in a tumour containing fully sensitive and mildly VCR-resistant cells only before treatment. The resistant cells made up 15% of the initial cell population and the population dynamics were driven by strategy A (panel **a**), strategy B (panels **b** and **d**), or strategy C (panel **c**). S denotes fully sensitive cells; O-mild, mildly VCR-resistant cells; O-strong, strongly VCR-resistant cells; C-mild, mildly CPM-resistant cells; C-strong, strongly CPM-resistant cells; and total, total cell population. MTD represents the total cell population responding to the default schedule: applying VCR and CPM at their MTDs for eight cycles (2688 h). VCR and O both denote vincristine, while CPM and C both denote cyclophosphamide. These trajectories were obtained by solving the model numerically with the ode45 solver in MATLAB.

The optimal schedules for mixtures of fully sensitive and mildly VCR-resistant cancer cells are shown in Figure 2 (O-mild rows). In these cases, we initialised the clonal compositions so that only $n_{0,0}$ and $n_{1,0}$ were non-zero. Using CPM at its MTD throughout the induction phase was found to be optimal in these cases. The optimal schedules all have nine cycles and start without VCR before applying it at its MTD from the third cycle onwards. Figure 3a presents the population dynamics of the nine clones induced by the optimal schedule corresponding to the third O-mild row (15%) in Figure 2.

We can explain the results summarised above in terms of clonal competition. Our calibrated parameters and the experimental data we used for calibration [27,28] indicate that the resistant clone has a lower proliferation rate (drug resistance lowers fitness when the drug is absent): resistance to VCR, which confers no advantage on the resistant cells in the absence of VCR, comes at the expense of growth. When a population comprises fully sensitive and VCR-resistant cells, using CPM at its MTD fully exploits the sensitive clone, which is fitter than the VCR-resistant clone in the presence of CPM, to stop the VCR-resistant clone from expanding, thus helping CPM kill the VCR-resistant cells. As the VCR-resistant clone shrinks and the initially sensitive clone acquires resistance to CPM, the latter becomes VCR-sensitive only (not fully sensitive) and occupies an increasingly large fraction of the population, so introducing VCR will shrink the now dominant (if not fixated) VCR-sensitive clone in the population.

We carried out a sensitivity analysis of the five optimal schedules presented in Figure 2 (O-mild rows). We applied each optimal schedule to all five initial clonal compositions and found almost identical results for each composition. This is significant for two reasons. First, although the five optimal schedules have very similar doses, it is still desirable to minimise the total amount of VCR used (due to toxicity). Second, it is difficult to determine the size of the VCR-resistant clone precisely, so the robustness of these optimal schedules mitigates this experimental limitation.

The optimal schedules for mixtures of fully sensitive and strongly VCR-resistant cancer cells are shown in Figure 2 (O-strong rows). In these cases, we initialised the clonal compositions so that only $n_{0,0}$ and $n_{2,0}$ were non-zero. The results are similar to those discussed above (O-mild rows). One new feature is the trend that as the strongly resistant clone’s size increases, VCR appears slightly later in the optimal schedules. Another new feature is that the schedules are three cycles longer.

We can explain the new features in terms of the same mechanism we just proposed. This mechanism relies on the fully sensitive clone’s selective advantage over the VCR-resistant clone. Although CPM kills both, the sensitive clone will eventually acquire resistance to CPM, become VCR-sensitive only, and dominate the population. At this point, VCR will be effective against the dominant VCR-sensitive clone. Therefore, if the VCR-resistant clone initially occupies a larger fraction of the population, it will take more cycles to shrink it, meaning it will be desirable to retain the competing clone a bit longer. This is in theory also relevant to a mixture of fully sensitive and mildly VCR-resistant cells and can indeed be observed in the first five rows in Figure 2 (O-mild rows). In reality, this trend is clearer for fully sensitive and strongly VCR-resistant cells because of a further effect. Regardless of its size, if the VCR-resistant clone resists VCR more strongly, it will be less fit in the presence of CPM and it will be desirable to exploit the clonal competition longer, but the initially sensitive clone will end up occupying a larger fraction of the population, so it will take more cycles of VCR treatment to shrink it when it is VCR-sensitive only in the second stage.

As with the previous set of results, we carried out a sensitivity analysis of the five optimal schedules presented in Figure 2 (O-strong rows). We applied each optimal schedule to all five initial clonal compositions. As the five optimal schedules are very similar, we unsurprisingly found five very similar outcomes for each initial clonal composition (differences less than 3%).

The top rows in Table 2 compare the results of applying the optimal schedules with those achieved using the default schedule. One can observe the uniformly positive gains relative to the default schedule.

### 3.2. Mixtures of Fully Sensitive and CPM-Resistant Cells

In these cases, we initialised the clonal compositions so that only $n_{0,0}$ and $n_{0,1}$ were non-zero. The five optimal schedules found for mixtures of fully sensitive and mildly CPM-resistant cancer cells are identical. This optimal schedule applies VCR and CPM at their MTDs for six cycles. Figure 3c presents the population dynamics of the nine clones induced by the optimal schedule (MTDs for six cycles) found for a tumour wherein 15% of the cells are mildly CPM-resistant.

We can explain the ineffectiveness of the clonal competition implied by this optimal schedule in terms of the asymmetry in the drugs’ cytotoxicities. It is true that in a mixture of CPM-resistant and fully sensitive cells, the addition of VCR will favour the sensitive cells over the CPM-resistant cells. Our calibrated parameters and the experimental data we used for calibration [27,28] indicate that the resistant clone has a lower proliferation rate: resistance to CPM, which confers no advantage on the resistant cells in the absence of CPM, comes at the expense of growth. However, VCR is less cytotoxic than CPM, so the advantage of clonal competition is nullified by the loss of CPM’s cytotoxic action in this situation. In other words, even if the sensitive clone can help by suppressing the CPM-resistant clone in the absence of CPM, VCR will still fail to shrink the suppressed clone sufficiently. Therefore, the optimal schedule recommends the use of both drugs at their MTDs to shrink the sensitive clone quickly and before it becomes resistant to CPM, and switch to another therapy when the total population is presumably reduced, fragmented, and vulnerable to a second strike. This strategy works because a mildly resistant tumour shrinks rapidly in the presence of both drugs at their MTDs.

The optimal schedules for mixtures of fully sensitive and strongly CPM-resistant cancer cells are shown in Figure 2 (C-strong rows). In these cases, we initialised the clonal compositions so that only $n_{0,0}$ and $n_{0,2}$ were non-zero. All the optimal schedules consist of 12 cycles and apply VCR before adding CPM in the last few cycles. In all of them, the VCR doses are mostly below the MTD before the eighth cycle. The optimal schedule for a tumour with a large CPM-resistant clone introduces CPM later. Figure 3b presents the population dynamics of the nine clones induced by the optimal schedule corresponding to the third C-strong row (15%) in Figure 2.

We can explain the differences between these optimal schedules and those obtained for mixtures of fully sensitive and mildly CPM-resistant cells in terms of clonal competition and cytotoxic levels. Like the scenario we just discussed, VCR is not as cytotoxic as CPM, but the higher level of resistance to CPM in this scenario also means the strongly resistant cells are even less fit than the mildly resistant cells in the presence of VCR. Our calibrated parameters and the underlying experimental results [27,28] indicate that the strongly resistant clone has a lower proliferation rate: resistance to CPM, which confers no advantage on the resistant cells in the absence of CPM, comes at the expense of growth. In this scenario, the advantage of clonal competition more than compensates for the loss of CPM’s cytotoxic action. Using low VCR doses in the early cycles can keep the sensitive clone in the population longer to prolong clonal competition, thereby giving VCR more time to shrink the suppressed CPM-resistant clone. For the same reason, if the resistant clone occupies more of the initial cell population and needs more time to respond to VCR, CPM should be introduced later. As the initially fully sensitive clone also has more time to dominate the population in the first stage, more cycles of CPM treatment are needed to shrink it in the second stage, hence the need for 12 cycles.

We carried out a sensitivity analysis of the optimal schedules presented in Figure 2 (C-strong rows) by applying each to all five initial clonal compositions. According to the results, depending on the initial clonal composition, applying a wrong schedule can increase the cell population’s final size by up to 15%.

The middle rows in Table 2 compare the results achieved using the optimal schedules with those achieved using the default schedule. The schedules we found during optimisation are superior, as expected.

### 3.3. Mixtures of Fully Sensitive, VCR-Resistant, and CPM-Resistant Cells

The optimal schedules for mixtures of mildly VCR-resistant ($n_{1,0}$), mildly CPM-resistant ($n_{0,1}$), and fully sensitive ($n_{0,0}$) cells are very similar (both mild rows in Figure 2). They all apply both drugs at their MTDs, for seven cycles if the two resistant clones constitute 5% of the population and six cycles if more. In a sensitivity analysis, we applied both (six and seven cycles) to a virtual tumour wherein resistant cells made up 5% of the initial population. We found almost identical results (difference around 3%). Since it is desirable to minimise toxicity, we conclude that applying both drugs at their MTDs for six cycles is always optimal for mixtures of mildly VCR-resistant, mildly CPM-resistant, and fully sensitive cells.

The optimal schedules for mixtures of strongly VCR-resistant ($n_{2,0}$), strongly CPM-resistant ($n_{0,2}$), and fully sensitive ($n_{0,0}$) cells were found to vary qualitatively with the precise clonal composition (both strong rows in Figure 2). If the resistant clones occupy 10% of the population or less, the optimal schedules apply both drugs at the MTDs for four cycles. Otherwise, the optimal schedules have 12 cycles, apply VCR at the MTD throughout, and use CPM at the MTD in the last six cycles. Figure 3d shows the population dynamics of the nine clones induced by the optimal schedule corresponding to the second both strong row in Figure 2, found for a tumour wherein 15% of the cells are resistant. According to our sensitivity analysis, applying an optimal schedule to a clonal composition in the wrong category can increase the cell population’s final size by up to 50%.

The optimal schedules for mixtures of all cells without cross-resistance ($n_{0,0}$, $n_{1,0}$, $n_{0,1}$, $n_{2,0}$, and $n_{0,2}$) display a similar qualitative change (all clones rows in Figure 2). If the resistant clones occupy 20% of the population or less, they apply both drugs at their MTDs for four (10%, 15%, and 20%) or five (5%) cycles. If they constitute 25% of the population, the optimal schedule has 12 cycles, with VCR applied at the MTD throughout, and uses CPM at its MTD in the last seven cycles. We carried out a sensitivity analysis and found that applying an optimal schedule to a clonal composition in the wrong category will increase the cell population’s final size significantly: up to 60%.

The patterns in these schedules are very similar to those in the schedules optimised for mixtures of CPM-resistant and fully sensitive cells. The strategy depends on the balance between CPM’s superior cytotoxic function and the sensitive clone’s ability to suppress the CPM-resistant clone in the absence of CPM. If the CPM-resistant clone resists CPM strongly enough and it occupies a sufficiently large part of the tumour, the latter factor (clonal competition) will confer a greater advantage than CPM’s superior cytotoxic function. In this case, the strategy is to apply VCR before CPM. On the other hand, if the CPM-resistant clone is only mildly resistant or if it is strongly resistant but too small, then the advantage conferred by clonal competition cannot compensate for the loss of CPM’s cytotoxic action. In this case, the strategy is to apply both drugs at their MTDs to eradicate the sensitive clone and before it becomes resistant to CPM, and switch to a different therapy when the population is presumably reduced, fragmented, and vulnerable to a second strike. The second strategy works because a mildly resistant tumour shrinks rapidly in the presence of both drugs at their MTDs.

The bottom rows in Table 2 compare the results obtained using the optimal schedules with those obtained using the default schedule. We can observe that the use of evolutionary principles can improve the default schedule.

## 4. Discussion

In 2010, the neuroblastoma community first proposed the inclusion of systems modelling in neuroblastoma research and management [41]. At the time of writing, while the literature does record detailed models of the intracellular dynamics pertinent to neuroblastoma [42,43,44,45], mechanistic modelling at the population level is slightly behind. In one study, ordinary differential equations were used to model the pharmacokinetics and pharmacodynamics of bevacizumab, vascularisation, and neuroblastoma growth [46]. In another study [47], a semi-mechanistic approach reducing the metastatic process to two basic phenomena—growth and dissemination—was used, but the influence of drugs was neglected. A recent publication reports the use of dynamical systems theory to reveal the relationship between neuroblastoma cells, the immune system, and the oncolytic adenovirus ICOVIR-5 within the context of Celyvir, a kind of oncolytic virotherapy [48]. Another trend centres around the development of a digital twin, empowered by multi-scale modelling and imaging biomarkers [49,50,51,52,53].

There are two major gaps in the literature. First, no one has attempted to exploit evolutionary principles to steer neuroblastoma progression at the population level. Second, optimising traditional chemotherapy is a cost-effective way to improve neuroblastoma treatment as the therapeutic agents have already been approved, but the one-size-fits-all rapid COJEC regimen is still the standard. The most relevant studies were published over a decade ago [54,55]. In both studies, only topotecan was considered, and they focused on myelosuppression and cell cycle–specific mechanisms, not evolutionary dynamics and drug resistance mechanisms. These issues deserve more attention, as evolutionary approaches have been shown to prolong progression-free survival in breast cancer [56] and castration-resistant prostate cancer [57] patients.

In this paper, we have presented a potentially significant stride in this direction for neuroblastoma, similar to a recent theoretical study [58] and other previous ones relating to other cancer types such as pancreatic, colorectal, and melanoma cancers [59].

### 4.1. Therapeutic Strategies Based on General Evolutionary Principles

Generalising from the schedules optimised for VCR and CPM, we propose three therapeutic strategies. Each is potentially applicable when two conditions are met. First, there are two drugs whose cytotoxic activities are significantly different. Second, a cancer cell genetically resistant to one drug is always less fit than a sensitive cancer cell in the absence of this drug: resistance comes at the expense of growth. Our calibrated parameters indicate that these two conditions are met in the case of treating neuroblastoma with VCR and CPM.

Provided that these two conditions are met, the proposed strategies form a classification scheme based on a series of four questions about the tumour to be treated.

1 Is the tumour to be treated already resistant to the less cytotoxic drug but not the other drug? If so, the optimal strategy is to apply the more effective drug at its MTD to exploit clonal evolution to kill the resistant clone effectively before adding the other drug to the regimen to shrink the tumour, which is mostly sensitive to the less effective drug at the end of the first stage. Finally, switch to a third drug (or another intervention) to exploit the final state of the tumour. This is strategy A. For instance, strategy A was found to be optimal for mixtures of fully sensitive and mildly VCR-resistant cancer cells (O-mild rows in Figure 2). Figure 3a presents the population dynamics of the nine clones induced by the optimal schedule corresponding to the third O-mild row (15%) in Figure 2.
2 If the tumour is already resistant to the more cytotoxic drug only, is it mildly or strongly resistant to it? If it is strongly resistant, a similar two-stage strategy will work, but only the less effective drug is used in the first stage, while both drugs are used in the second stage. Furthermore, both stages should last longer than in strategy A to prolong clonal competition, thus maintaining a negative selection pressure on the resistant clone. This change is necessary because resistance to the more cytotoxic drug is harder to deal with. This is strategy B. For instance, strategy B was found to be optimal for mixtures of fully sensitive and strongly CPM-resistant cancer cells (C-strong rows in Figure 2). Figure 3b presents the population dynamics of the nine clones induced by the optimal schedule corresponding to the third C-strong row (15%) in Figure 2. During the dynamic simulation’s first stage, the strongly CPM-resistant clone (orange line) shrank even though the whole population (black line) and the fully sensitive clone (grey line) grew. If the tumour is mildly resistant to the more cytotoxic drug only, the optimal strategy is to use both drugs at their MTDs for a short duration to shrink the sensitive clone in the tumour and then switch to a third drug (or another intervention) targeting the presumably reduced and fragmented cell populations. This is strategy C. For instance, strategy C was found to be optimal for mixtures of fully sensitive and mildly CPM-resistant cancer cells (C-mild row in Figure 2). Figure 3c presents the population dynamics of the nine clones induced by the optimal schedule corresponding to the C-mild row in Figure 2; the resistant clone made up 15% of the initial population in this simulation.
3 If the tumour is already resistant to both drugs, is it mildly or strongly resistant to the more cytotoxic drug, or are there both mildly and strongly resistant cells? If the tumour is only mildly resistant, strategy C is recommended.
4 If the tumour is strongly resistant or contains both mildly and strongly resistant cells, what is the total fraction of cells that are resistant? A low fraction favours strategy C, while a high fraction favours strategy B. As a rule of thumb, based on the results presented in Figure 2, a fraction below 15% is considered low in a strongly resistant tumour and a fraction below 25% is considered low if the tumour contains both mildly and strongly resistant cells. For instance, strategy B was found to be optimal for the case where strongly VCR-resistant and strongly CPM-resistant cells constitute 15% of the initial population. Figure 3d shows the population dynamics of the nine clones induced by the optimal schedule corresponding to the second and last both strong rows in Figure 2; the resistant cells made up 15% of the initial population in this simulation. In the first stage, the CPM-resistant clone (orange line) was suppressed by the other clones in the presence of VCR only, but the VCR-resistant clone (blue line) expanded aggressively to dominate the population. In the second stage, the tumour dominated by the VCR-resistant clone responded to CPM effectively.

Suitable tactics would be needed to make each strategy work in practice. For strategy A or B, a key piece of information is the size of the clone resistant to the second drug. The larger it is, the longer both stages should last, as prolonging the first stage will give the evolutionarily advantaged clone more time to dominate the population, but it will also take more time to shrink the tumour in the second stage. A higher level of resistance (strong rather than mild) also justifies this tactic. In fact, strategy B differs from strategy A for precisely this reason. If strategy B is chosen, another relevant piece of information is whether the tumour has cells that are resistant to both drugs. If the tumour can only resist the more cytotoxic drug before treatment (harder to deal with), the less cytotoxic drug should be applied below its MTD in the first stage, thus keeping the sensitive clone in the population and a negative selection pressure on the resistant clone longer. If strategy C is chosen, the key decision is when to switch to the third drug (or another intervention). It is likely that the decision is highly sensitive to the parameters (cytotoxic functions and fitness levels) and initial conditions (clonal composition) characterising the tumour. In the absence of such data, our results presented in Figure 2 suggest that half the default length is a suitable estimate.

### 4.2. Clinical Translation

In practice, our theoretical results are only clinically applicable if there are data to answer the above questions and inform the decisions. For example, the clonal composition of a tumour is rarely, if ever, known in a clinical setting. Therefore, we need to combine our theoretical results with emerging medical technologies. In other words, we need to build a decision support system to realise our vision of a personalised multi-modal therapy for high-risk neuroblastoma.

While the cited study [59] found exploitable principles for the case where there are no single mutations conferring resistance to two drugs, we discovered evolutionary principles that are useful when there are two differentially cytotoxic drugs, and each drug can only be resisted at the expense of growth (drug resistance lowers fitness when the drug is absent). We integrated the principles we discovered within a conceptual framework. The current standard of care, the one-size-fits-all multi-modal therapy for high-risk neuroblastoma, begins with induction chemotherapy, followed by surgical removal of the tumour and consolidated by myeloablative chemotherapy with autologous haematopoietic stem cell transplantation, and finishes with maintenance therapy involving anti-GD2 monoclonal antibodies, cytokines, and isotretinoin. Our results are potentially useful within this familiar context because the classification scheme can optimise the induction phase, as long as there are data to inform the decisions involved.

On the other hand, our results also imply that the neuroblastoma community must not adopt the generalising approach used in our study, which considered two drugs only. According to our mini-review in the Supplementary Materials file, there are at least 16 chemotherapeutic agents in use for the neuroblastoma treatment, in addition to isotretinoin and anti-GD2 monoclonal antibodies. If we expand this study to more than two drugs, the number of clones and the number of parameters will both increase exponentially, although cross-resistance can potentially allow some parameters to be lumped together. As the model becomes increasingly sensitive to its initial conditions and interminable bifurcations emerge, a generalising approach will quickly become impractical. Even the general principles presented herein require patient-specific details to work in practice, most notably the precise clonal composition of a tumour before treatment. Therefore, the proposed decision support system must consider all 16 agents, the mutations and oncogenic pathways enriched and activated by them [60], and an individual tumour’s clonal composition as determined by multi-region sequencing and liquid biopsies [60]. It will personalise and improve the one-size-fits-all multi-modal therapy by optimising chemotherapy schedules, streamlining other steps (surgery and immunotherapy), and leveraging the targeted therapies mentioned in the introduction [18,19].

### 4.3. Design Choices

It is possible that the neuroblastoma cells do not just compete for resources but also synergise by enhancing nutrient transport. Their interactions with healthy cells such as Schwann cells [61] and the microenvironment [62] are also interesting. However, in this instance, we decided to abstract these phenomena away because they would have required spatial modelling. To be consistent with the observation that neuroblastoma cells are plastic and can undergo a noradrenergic-to-mesenchymal transition to resist chemotherapy more effectively [6], we chose to model phenotypic adaptation in addition to genetically conferred resistance. Our model assumes that it is a continuous mechanism that activates at a constant rate as long as the drug concentration is above a minimum level. However, in practice, a higher dose will remain in the system longer, thus maintaining a drug concentration above the minimum level longer. Therefore, the degree of phenotypic adaptation (resistance) does go up with the dose.

Another important modelling choice we made concerned mutation. In our model, the neuroblastoma cells in a clone can only mutate into a neighbouring clone defined by Figure 1. Furthermore, the same mutation rate applies in both directions between two neighbouring clones. This is consistent with the biological reality that random mutations can make a cell grow faster, resist drugs more effectively, be more susceptible to apoptosis, and behave in other contradictory ways. Each mutation can induce one or more of these effects, or may do nothing at all. In our model, clonal evolution is driven by the environmental pressures exerted by the two drugs and phenotypic adaptation, which shifts the balance between drug resistance and cell growth. These biased selective pressures act on the unbiased random mutations.

We decided to evaluate vincristine and cyclophosphamide in this pilot study because they belong to two different categories of drugs. While vincristine is a plant alkaloid which disrupts the M phase, cyclophosphamide is an alkylating agent which acts throughout the cell cycle. As they kill cancer cells with different mechanisms, we considered them together in the spirit of finding evolutionary traps.

The biggest obstacle encountered in this study was the lack of suitable human-derived data for calibration and validation. To circumvent the problem, we found and aggregated mostly in vitro experimental data from different sources.

Due to the large number of optimisation problems and the high computational costs associated with the genetic algorithm, we decided to use a computationally efficient population-based modelling approach instead of the multi-type branching process used elsewhere [59]. In each optimisation problem, we had up to 24 control variables. To counter the curse of dimensionality [63], which would have required a vast amount of data to explore the solution space thoroughly, we used a genetic algorithm to eliminate the less promising regions.

### 4.4. Validity and Future Work

We are confident that our results are qualitatively valid, potentially useful, and reasonably robust. Despite the assumptions we made and the difficulty of calibrating our model, we successfully used it to simulate realistic population dynamics, reproducing experimentally observed trends. The evolutionary principles revealed by us are consistent with the theoretical insights in the literature [12,58]. We integrated them in a classification scheme: concrete steps of applying these principles to treat a neuroblastoma patient in a clinical setting. While the general motivation for introducing evolutionary principles to chemotherapy is to overcome drug resistance, our results highlight the benefit of exploiting clonal competition in particular. Earlier in the manuscript, while presenting our results, we explained how our proposed strategies work from a mechanistic perspective. In the case of strategy C, our results suggest that the use of evolutionary principles can reduce the number of cycles to minimise the unpleasant toxicological side effects of chemotherapy. Finally, we found that the optimal chemotherapy schedules are mostly robust even when the clonal composition in not precisely known.

Notwithstanding these positives, the model can be improved upon—in addition to the extensions discussed within the context of the decision support system—by considering neuroblastoma’s spatial architecture, as it has been shown to be an important factor in adaptive therapy [58,64]. Specifically, as discussed above, a spatial model can describe the synergistic effects abstracted out in our model, such as the spatial patterns arising from the interactions between neuroblastoma and Schwann cells [61], and the tumour microenvironment [62]. It can also compare the clearance rates of different tumours. Another potential improvement is to complement the population-based model with an agent-based model of the cellular scale, which will be useful for evaluating the impact of stochastic perturbations on the small, fragmented, and genetically homogeneous cell populations emerging from the induction phase.

## 5. Conclusions

We have designed and calibrated a mathematical model to describe the non-linear phenomena within neuroblastoma tumours responding to vincristine and cyclophosphamide, including growth, clonal competition, mutation, plastic response, drug delivery, and drug-induced cell death. By coupling the model to a bespoke optimisation algorithm, we found a broad range of optimal chemotherapy schedules minimising the final size for a broad range of tumours with different genetic pre-treatment compositions. Our results point to evolution-based strategies to exploit the cytotoxic difference between two drugs in a patient-specific manner. We translated our new theoretical insights into concrete steps presented within a classification scheme. In addition to being qualitatively valid and reasonably robust despite the assumptions made, the results suggest that the multi-agent rapid COJEC regimen can be personalised in a similar manner. Beyond induction chemotherapy, the results and tools presented herein lay the foundation of a personalised therapy leveraging targeted therapies and emerging medical technologies such as liquid biopsies, to improve today’s one-size-fits-all multi-modal protocol.

## Figures and Tables

**Table 1:** Parameters obtained by model calibration.

Parameters	Values	Units	Meanings
$z_{1}$	0.91	h ${}^{-1}$	VCR clearance rate
$z_{2}$	0.12	h${}^{-1}$	CPM clearance rate
$r_{0,0}$	$8.5\cdot 10^{-3}$	h${}^{-1}$	Sensitive clone’s growth rate
$r_{1,0}$	$7.7\cdot 10^{-3}$	h${}^{-1}$	VCR-10 clone’s growth rate
$r_{2,0}$	$7.5\cdot 10^{-3}$	h${}^{-1}$	VCR-20 clone’s growth rate
$r_{0,1}$	$7.7\cdot 10^{-3}$	h${}^{-1}$	CPM-20 clone’s growth rate
$r_{0,2}$	$7.5\cdot 10^{-3}$	h${}^{-1}$	CPM-32 clone’s growth rate
$r_{1,1}$	$7\cdot 10^{-3}$	h${}^{-1}$	VCR-10-CPM-20 clone’s growth rate
$r_{1,2}$	$6.8\cdot 10^{-3}$	h${}^{-1}$	VCR-10-CPM-32 clone’s growth rate
$r_{2,1}$	$6.8\cdot 10^{-3}$	h${}^{-1}$	VCR-20-CPM-20 clone’s growth rate
$r_{2,2}$	$6.6\cdot 10^{-3}$	h${}^{-1}$	VCR-20-CPM-32 clone’s growth rate
*K*	$10^{10}$	cells	Carrying capacity of the tumour
μ	$10^{-4}$	Dimensionless	Mutation rate
$\alpha_{1}$	$1.122\cdot 10^{4}$	Dimensionless	Shape parameter in mortality function (VCR)
$\beta_{1}$	0.6704	Dimensionless	Shape parameter in mortality function (VCR)
$m_{1}^{0,0}$	40.4	h${}^{-1}$	Sensitive clone’s maximum mortality rate due to VCR
$m_{1}^{0,1}$	6.8	h${}^{-1}$	VCR-10 clone’s maximum mortality rate due to VCR
$m_{1}^{0,2}$	6	h${}^{-1}$	VCR-20 clone’s maximum mortality rate due to VCR
$\alpha_{2}$	$2.9507\cdot 10^{-5}$	Dimensionless	Shape parameter in mortality function (CPM)
$\beta_{2}$	1	Dimensionless	Shape parameter in mortality function (CPM)
$m_{2}^{0,0}$	$3.1474\cdot 10^{-6}$	h${}^{-1}$	Sensitive clone’s maximum mortality rate due to CPM
$m_{2}^{0,1}$	$1.5737\cdot 10^{-6}$	h${}^{-1}$	CPM-20 clone’s maximum mortality rate due to CPM
$m_{2}^{0,2}$	$9.4422\cdot 10^{-7}$	h${}^{-1}$	CPM-32 clone’s maximum mortality rate due to CPM
nuovo$T_{min}$	0	days	nuovoMinimum memory period associated with phenotypic adaptation
$T_{max}$	10	days	nuovoMaximum memory period associated with phenotypic adaptation
nuovo$\Phi_{1_{min}}$	0	Dimensionless	nuovoMinimum effect of phenotypic adaptation on growth
$\Phi_{1_{max}}$	1	Dimensionless	Maximum effect of phenotypic adaptation on growth
nuovo$\Phi_{2_{min}}$	0	Dimensionless	nuovoMinimum effect of phenotypic adaptation on drug-induced mortality
$\Phi_{2_{max}}$	2	Dimensionless	Maximum effect of phenotypic adaptation on drug-induced mortality

**Table 2:** Results of applying the optimal chemotherapy schedules for mixtures of fully sensitive and VCR-resistant cells, presented in the top rows (below VCR size); mixtures of fully sensitive and CPM-resistant cells, presented in the middle rows (below CPM size); and mixtures of fully sensitive, VCR-resistant, and CPM-resistant cells, presented in the bottom rows (below Both size). The cell count (N), final size (FS), and gain (G) columns show the final cell count achieved with the optimal schedule for each initial clonal composition, the final size as a percentage of the initial size ($5\cdot 10^{9}$), and the gain relative to using MTDs for eight cycles (achieving $N_{MTD}$), respectively. The gain refers to the percentage difference in tumour size: $(\frac{N_{MTD}-N}{N_{MTD}})\times 100$. Each size (5, 10, 15, 20, and 25%) refers to the fraction of the initial population occupied by resistant cells. The strongly VCR-resistant cells were killed more effectively than the mildly VCR-resistant cells in the simulations because the strategy of exploiting clonal competition worked more effectively against the strongly resistant cells. Resistance to VCR comes at the expense of growth in the absence of VCR in our model.

VCR Size	Mild			Strong					
	N ($\cdot 10^{8}$)	FS (%)	G (%)	N ($\cdot 10^{8}$)	FS (%)	G (%)			
5%	1.17	2.34	16.09	0.53	1.06	50.48			
10%	1.80	3.6	21.18	0.85	1.7	50.29			
15%	2.38	4.76	21.40	1.11	2.22	51.35			
20%	2.91	5.82	21.07	1.36	2.7	51.00			
25%	3.42	6.84	19.46	1.60	3.2	50.25			
**CPM Size**	**Mild**			**Strong**					
	**N ($\cdot 10^{8}$)**	**FS (%)**	**G (%)**	**N ($\cdot 10^{8}$)**	**FS (%)**	**G (%)**			
5%	4.21	8.42	8.40	9.93	19.86	61.40			
10%	6.70	13.4	10.10	14.00	28	55.89			
15%	8.70	17.4	8.58	16.67	33.34	51.57			
20%	10.33	20.66	6.65	19.20	38.4	46.59			
25%	11.69	23.38	4.79	21.60	43.2	41.49			
**Both Size**	**Mild**			**Strong**			**Mild and Strong**		
	**N ($\cdot 10^{8}$)**	**FS (%)**	**G (%)**	**N ($\cdot 10^{8}$)**	**FS (%)**	**G (%)**	**N ($\cdot 10^{8}$)**	**FS (%)**	**G (%)**
5%	3.04	6.08	1.31	7.67	15.30	58.60	5.42	10.84	45.06
10%	4.92	9.84	4.30	11.38	22.76	55.02	8.14	16.28	47.17
15%	6.40	12.8	4.82	12.27	24.24	57.42	10.05	20.1	46.31
20%	7.72	15.44	4.01	12.53	25.06	59.58	11.83	23.66	43.90
25%	8.81	17.62	2.98	12.68	25.36	60.92	12.12	24.24	46.95

## Data Availability

The datasets used for calibration and validation are available in the literature. Please refer to the cited articles. The code to simulate clonal evolution during chemotherapy and to implement the genetic algorithm is available in a GitHub repository (accessed on 25 March 2023): https://github.com/kywertheim/OptimiseChemoNeuroblastoma.

## Supplementary Materials

The following are available online at https://www.mdpi.com/article/10.3390/cancers15071986/s1; Section S1: Model Structure. Section S2: Drugs Approved for Neuroblastoma Treatment. Section S3: Model Calibration. Section S4: Model Validation. Section S5: Optimising Chemotherapy Schedules. Figures S1: Saturation kinetics of sensitive neuroblasts responding to vincristine (VCR). In an experiment, sensitive neuroblasts were added to cultures with different concentrations of VCR and in each case, the extent of cell death was measured after four hours. We used our calibrated model to reproduce the experiment. This figure reports, for each VCR concentration, the extent of cell death at equilibrium. Figure S2: Saturation kinetics of sensitive neuroblasts responding to cyclophosphamide (CPM). In an experiment, CPM was used to treat neuroblastoma in a transgenic murine model for 24 h. Different CPM concentrations were used. We used our calibrated model to reproduce the experiment. This figure reports, for each CPM concentration, the extent of cell death at equilibrium. Figure S3: Clonal evolution driven by multiple cycles of treatment with cyclophosphamide (CPM). In an experiment, Th-MYCN genetically engineered mice were exposed to multiple CPM doses and changes in tumour size were tracked in 13 mice. We averaged the 13 drug schedules to obtain one representative schedule. In each of its first four cycles, 16 mg kg${}^{-1}$ of CPM was given. In the next three cycles, a higher dose (24 mg kg${}^{-1}$) was used. In the last four cycles, the dose was increased further to 32 mg kg${}^{-1}$. Each cycle lasted 164 h. We used our calibrated model to simulate how CPM-resistant clones had emerged in the experiment. CPM was administered in the first hour of each cycle. Figure S4: Simulated population sizes in cultures with different neuroblastoma cell lines with and without vincristine (VCR). The experimental study generated VCR-resistant neuroblastoma cell lines by culturing neuroblastoma Be2c cells with VCR at gradually increasing concentrations. The growth rates of sensitive and VCR-20 (strongly VCR-resistant) cells cultured with and without VCR were measured. First, the green bar pertains to untreated sensitive cells, while the dark green bar pertains to sensitive cells treated with 20 ng mL${}^{-1}$ of VCR for 48 h. Second, the grey bar is about VCR-20 cells kept in a medium with VCR. Third, although both the blue and dark blue bars describe VCR-20 cells cultured for three weeks in a VCR-free medium, the cells described by the latter were treated with 20 ng mL${}^{-1}$ of VCR for another 48 h. In each panel, the black line represents the number of cells. Figure S5: Demonstration of multidrug resistance in simulations. In an experiment, various cyclophosphamide (CPM)-resistant cell lines were treated with vincristine (VCR) at different concentrations. We used the calibrated model to reproduce the study. This figure reports the equilibria reached by sensitive (green line) and CPM-resistant (red line) cells in simulations at different VCR concentrations.
