# Supplementary material for: Mathematical Model of Clonal Evolution Proposes a Personalised Multi-Modal Therapy for High-Risk Neuroblastoma
Source: Cancers (Basel). 2023 Mar 26;15(7):1986. doi: 10.3390/cancers15071986 (PMC10093626; doi:10.3390/cancers15071986)
Supplement: Supplementary file 1 [file cancers-15-01986-s001.zip › cancers-2226123-supplementary.pdf]

Supplementary Materials

Matteo Italia[1], Kenneth Y. Wertheim[2,3,4,5], Sabine Taschner-Mandl[6], Dawn Walker[2,3], and Fabio Dercole[1]

[1]Department of Electronic, Information, and Bioengineering, Politecnico di Milano, Milano, Italy.

[2]Insigneo Institute for *in Silico* Medicine, University of Sheffield, Sheffield, the UK.

[3]Department of Computer Science, University of Sheffield, Sheffield, the UK.

[4]Centre of Excellence for Data Science, Artificial Intelligence, and Modelling, University of Hull, Kingston upon Hull, the UK.

[5]School of Computer Science, University of Hull, Kingston upon Hull, the UK.

[6]St. Anna Children's Cancer Research Institute, Vienna, Austria.

matteo.italia@polimi.it

S1 Model Structure

As described in the quick guide to our methodology, our model considers the population dynamics of nine clones evolving under the influence of two chemotherapeutic drugs. Each clone, due to genetic mutations, has two resistance levels specific to the two drugs, which are represented as a discrete pair: $i, j \in R = \{0, 1, 2\}$.

The clones compete for space and resources to support their logistic growth, interconvert by mutation during cell division, and die due to chemotherapy. As illustrated by the arrows in Fig.1, each clone can only mutate to the clones that differ by one resistance level with respect to one drug.

The quick guide omits the details regarding phenotypic adaptation and the mortality rates' dependence on drug concentrations. The purpose of this section is to supplement the quick guide with these details.

Phenotypic adaptation. When cancer cells are under stress imposed by chemotherapeutic agents, they can develop temporary multidrug resistance, such as the expression of efflux pumps to remove multiple drugs via an ATP-dependent mechanism [1].

Phenotypic adaptation is a delayed mechanism in our model. Each time point is associated with a 'memory period': the previous T_{max} hours. It is also associated with a 'memory level': $\tau \in [\mathrm{T}_{min}, \mathrm{T}_{max}] = [0, \mathrm{T}_{max}]$. If $\tau = \mathrm{T}_{min} = 0$, it means chemotherapy was inactive at least in the previous T_{max} hours. If $\tau = \mathrm{T}_{max}$, it means chemotherapy was active throughout the entire 'memory period' (at least in the previous T_{max} hours). If $c_1 + c_2 > 0.001$—c_d denotes the concentration of drug d—at a time point during the 'memory period', chemotherapy was active at that point. It simply means that as long as the constraint that $c_1(t) + c_2(t) > 0.001$ is satisfied at a point time t, chemotherapy is considered active at that point regardless of the precise drug concentrations. This particular threshold, as opposed to zero, was chosen because neither c_1 nor c_2 will ever be zero in a simulation unless it is initially zero. However, the higher the drug dose is, the longer the constraint will be met, leading to more effective phenotypic responses.

As these pumps cost energy in the form of ATP, any resulting decline in drug-induced death rates must come at the expense of biomass production and growth [2]. Mathematically, ϕ_1 and ϕ_2 are continuous functions that increase linearly with τ from $\Phi_{1_{min}} = \Phi_{2_{min}} = 0$ ($\tau = \mathrm{T}_{min} = 0$), corresponding to null phenotypic adaptation, to $\Phi_{1_{max}}$ or $\Phi_{2_{max}}$ ($\tau = \mathrm{T}_{max}$), corresponding to maximum phenotypic adaptation. Collectively, they model phenotypic adaptation.

Mortality rates. As a cell can only absorb a finite amount of drug in a finite time period, saturation will lead to diminishing returns on increases in the drug's concentration. Therefore, each mortality rate

is expressed as a non-linear function: $m_d^{i,j}(c_d) = \frac{m_{d,0}^{i,j} c_d}{1+\alpha_d c_d^{\beta_d}}$. The shape parameters, α_d and β_d, are dimensionless. Although c_d represents the drug concentration, we hereby declare that $\alpha_d c_d^{\beta_d}$ be dimensionless for the sake of simplicity. Simplistically, it means that c_d is divided by 1 unit of drug concentration before being exponentiated by β_d. The parameter $m_{d,0}^{i,j}$ (*per* unit of drug concentration *per* hour) is the maximum mortality rate when the drug concentration is far below its saturation level. Depending on the dose units we found in the literature, we adjusted the units of c_d and $m_{d,0}^{i,j}$ to ensure meaningful quantities were used during calibration, the optimisation studies, and the dynamic simulations.

S2 Drugs Approved for Neuroblastoma Treatment

The mathematical model proposed can be used to study any pair of drugs. Before choosing a pair for this study, we performed a literature review on the drugs approved for neuroblastoma treatment, as summarised below.

S2.1 Alkylating and DNA-crosslinking agents

Medical professionals have used alkylating agents, which act effectively on DNA throughout the cell cycle, to treat cancers for around eight decades [3].

Cisplatin is a platinum-containing drug which becomes active intracellularly and binds to the purine residues of DNA, thereby causing DNA damage and inhibiting DNA replication, ultimately triggering apoptotic cell death [4]. When it is used for neuroblastoma treatment, it is administered intravenously as an infusion over one to five days and is usually administered every 21 days [5, 6]. Remission has been observed after four to six cycles [6]. According to one study [7], a dosage comprising five doses of 40 mg m^{-2} is considered high. Neuroblastoma cells develop resistance to cisplatin by altering their expression of proteins involved in epithelial-to-mesenchymal transition (EMT), which also confers cross-resistance to temozolomide, etoposide, and irinotecan [8]. A study identified 139 genes upregulated by cisplatin-resistant neuroblastoma cells; they are involved in responses to stress and abiotic stimuli, metabolic regulation, apoptosis, proliferation, DNA repair, and catalysis [9].

Carboplatin is similar to cisplatin, but it is chemically less reactive and less toxic [10]. It is believed to interact with genomic DNA, tubulin, and other proteins [10]. Depending on the regimen, carboplatin has been combined with etoposide, cyclophosphamide, doxorubicin, and vincristine to treat neuroblastoma [11, 12]. According to a review [13], carboplatin resistance is not as understood as cisplatin resistance, but has similar mechanisms. The review lists the following as platinum resistance mechanisms: decreased blood flow to the tumour, extracellular conditions, reduced platinum uptake, increased efflux, intracellular detoxification by glutathione and others, decreased drug binding, DNA repair, decreased mismatch repair, defective apoptosis, anti-apoptotic factors, and quiescence [13].

Iproplatin, a recent addition to the family of platinum drugs, is also an analogue of cisplatin and behaves similarly, but it did not exhibit superior effectiveness to cisplatin or carboplatin in clinical trials [14]. It binds to DNA to form DNA crosslinks and platinum DNA adducts, thus inhibiting DNA replication and triggering apoptosis [15]. Although it is less prone to glutathione inactivation than cisplatin, tumour cells can resist it by repairing platination damage [15].

Cyclophosphamide is a nitrogen mustard which exerts its medicinal effects by alkylating DNA [16]. When it metabolises to an active form, it can inhibit protein synthesis and trigger programmed cell death by crosslinking with DNA and RNA [16]. As cyclophosphamide is immunosuppressive and selects for T cells, it can be used to eradicate malignant hematopoietic cells and immunomodulate regulatory T cells selectively [16]. This drug must undergo a cascade of metabolic events before it can exert its medicinal effects, so it is not surprising that the known resistance mechanisms centre around the disruption of this cascade by aldehyde dehydrogenase, gluthione, and glutathione S-transferase [17].

Busulfan kills cancer cells by disrupting DNA replication and its transcription to RNA, and by damaging DNA irreparably through guanine-adenine crosslinking, thus triggering apoptosis via the p53 pathway [18, 19]. A high-dose combination of busulfan and melphalan (BuMel regimen) is routinely used to treat high-risk neuroblastoma in Europe [20]. A gene expression analysis identified the cellular factors

underlying busulfan resistance, including their ability to evade cell cycle arrest in the G2 phase and apoptosis [21].

Thiotepa is a polyfunctional alkylating agent which can damage DNA via two pathways [22]. It has been used in tandem with BuMel before autologous stem cell transplantation in the treatment of very high–risk neuroblastoma [23]. CYP2B6 and 3A4 are human cytochrome P450s—key phase I enzymes that activate thiotepa–so mutations in their encoding genes may confer resistance to thiotepa on neuroblastoma cells [24].

Ifosfamide, an analogue of cyclophosphamide, belongs to the nitrogen mustard family [25]. Its metabolism, catalysed by CYP450 enzymes in the liver, produces metabolites that trigger apoptosis in a cancer cell by crosslinking with its DNA. The metabolites can also upregulate the reactive oxygen species (ROS), damaging the cell's DNA irreparably and inhibiting protein formation therein. Its toxicities include hemorrhagic cystitis, neurotoxicity, hematologic toxicity, nephrotoxicity, and more. As ifosfamide is an analogue of cyclophosphamide, it is unsurprising that their resistance mechanisms are similar [26].

Melphalan is another nitrogen mustard alkylating agent which works by alkylating guanine in DNA, thereby creating inter- and intrastrand crosslinks to inhibit DNA/RNA synthesis, triggering apoptosis [27]. In the treatment of high-risk neuroblastoma, two common regimens involving melphalan are BuMeL and CEM. The former combines melphalan with busulfan and the latter combines melphalan with carboplatin and etoposide [28]. According to an experiment on melphalan-resistant myeloma cells, enhanced interstrand crosslink repair via the FA/BRCA pathway is a key resistance mechanism [29].

Temozolomide, after intracellular hydrolysis, is a methylating agent which acts on DNA guanine bases and other nucleobases to trigger apoptosis [30]. Resistance to temozolomide in glioblastoma is largely due to MGMT, a protein which repairs temozolomide-induced DNA lesions, and DHC2, which transports DNA repair proteins to a cell's nucleus [31].

S2.2 Plant alkaloids with alternative mechanisms of action

These chemotherapeutic agents are derived from plants and unlike the DNA-crosslinking agents, their mechanisms of action arrest the cell cycle at the G0/G1 and G2/M checkpoints, resulting in DNA repair or apoptosis [32].

Vincristine is an extract from *Catharanthus roseus* [33]. It is commonly used in combination with other chemotherapeutic agents, such as topotecan, carboplatin, cisplatin, and most relevantly, cyclophosphamide [34–36]. It works by interfering with microtubules to disrupt the formation of mitotic spindles during the M phase of the cell cycle. Vincristine further promotes cell cycle arrest during the M phase by upregulating cyclin B and downregulating cyclin D. Separately, it activates apoptotic factors, including caspases 3 and 9. Overall, it inhibits proliferation and triggers apoptosis. Resistance to it is achieved by upregulating BRCA1 [37]; survivin via the ERK1/2, Akt, and NF-B signalling pathways [38]; and P-glycoprotein [39].

Tenisopide is a derivative of podophyllotoxin, a component of the ethanolic extracts from dried roots and rhizomes of the genus *Podophyllum* [40]. Since topoisomerases are essential for DNA replication, chromosome condensation, and chromosome segregation [41], this topoisomerase II inhibitor can damage DNA and prevent cancer cells from entering mitosis by arresting them in the late S or G2 phase [40]. Generally, resistance to topoisomerase II inhibitors involves both the use of multidrug transporters and enzymatic alterations that may nullify the inhibitors [42]. For example, activating NF-κB upregulates genes that confer protection against apoptosis [43].

Etoposide is like tenisopide, a derivative of podophyllotoxin [40] and a topoisomerase II inhibitor [41]. Etoposide and teniposide exhibit qualitatively identical activities, but teniposide is only administered intravenously and at lower doses [40]. The two analogues are resisted by the same mechanisms [42].

Topotecan is derived from camptothecin, an extract from the bark of *Camptotheca acuminata*, and it is a topoisomerase I inhibitor [44]. Although both topoisomerase I and topoisomerase II relax and untangle large strands of DNA to change its topological state, the former targets single strands, while the latter

targets double strands [45]. Topotecan disrupts this process in order to damage DNA and hinder DNA repair, ultimately triggering apoptosis in cancer cells [44]. Based on preclinical studies, the resistance mechanisms against topotecan are likely to involve drug efflux pumps, mutations in the gene encoding topoisomerase I, and alterations in the way cancer cells respond to the interactions between topotecan and topoisomerase I [46].

Irinotecan, another derivative of camptothecin and also a topoisomerase I inhibitor, acts similarly to topotecan [44]. The resistance mechanisms deployed by cancer cells against the two drugs are similar too [46].

S2.3 Antibiotics

Anticancer antibiotics are secondary metabolites produced by microorganisms like bacteria, fungi, and actinomycetes or higher animals and plants [47]. They are mainly peptides and anthraquinones which can be classified into anthracyclines, mitomycin, bleomycin, actinomycin, guanorycin and endiyne. Their anticancer effects arise from anti-proliferative, pro-apoptotic, and anti-EMT mechanisms. They can kill cancer cells throughout the cell cycle, including the G0 phase. On the other hand, they may also advantage cancer cells by disrupting intestinal microbiota, thus promoting chronic inflammation, altering metabolism, causing genotoxicity, and weakening immunity.

Doxorubicin is an anthracycline which can damage DNA throughout the cell cycle [48]. It does so by binding to DNA-associated enzymes (such as topoisomerases) and intercalating with the base pairs on the DNA double helix. As a result, cell cycle progression is arrested during the G1 and G2 phases, and apoptosis is triggered. It can also intercalate itself into DNA and generate free radicals to damage it. A major side effect of doxorubicin is cardiotoxicity, which leads to cardiomyopathy [49]. Resistance to it is achieved through the MAPK/ERK pathway, which can protect cancer cells from oxidative stress [50]; ABC transport proteins, which can pump drugs out of cancer cells [51]; and amplification of the *TOP2A* and *ERBB2* genes [51].

Epirubicin is also an anthracycline and actually a semi-synthetic derivative of doxorubicin [52]. Like other anthracyclines, its anticancer effects are thought to result from intercalation between DNA base pairs and stabilisation of the topoisomerase II-DNA complex, leading to irreversible DNA strand breakage. DNA lesions may lead to cell death throughout the cell cycle, although anthracyclines are most active during the S and G2 phases. Cellular resistance to epirubicin and other anthracyclines involves P-glycoprotein, changes in topoisomerase II activity, upregulation of glutathione and glutathione-S-transferase, induction of heat shock proteins, and blockade of apoptopic pathways.

S2.4 Others

Isotretinoin (retinoic acid) is used in the maintenance phase of the multi-modal therapy for high-risk neuroblastoma [53]. It works against neuroblastoma cells by inhibiting proliferation and inducing differentiation [54]. Although it improves the event-free survival rate significantly, it selects for neuroblastoma cells with MYCN upregulation, leading to resistance to the agent [55].

Dinutuximab is a monoclonal antibody produced using mouse cells, while dinutuximab beta is produced using hamster cells [56]. They are commonly used in combination with granulocyte-macrophage colony stimulating factor (GM-CSF), interleukin-2 (IL2), and isotretinon in the maintenance phase. They work by binding to the glycolipid GD2, which is expressed by neuroblastoma cells, to mobilise the immune system against the cancer cells [57]. These agents are actually not considered chemotherapeutic agents because they are antibodies acting against a specific molecular target (GD2); they fall under the broad category of targeted therapy, specifically immunotherapy. To the best of our knowledge, they are the only immunotherapeutic agents approved for neuroblastoma. Little is known about the resistance mechanisms acting against dinutuximab and dinutuximab beta, but neuroblastoma cells are known to create an immunosuppressive environment in the tumour [58].

S3 Model Calibration

After we reviewed all the approved drugs for neuroblastoma, the decision to evaluate vincristine (VCR, $d = 1$) and cyclophosphamide (CPM, $d = 2$) was made. We made this choice for three reasons. First, vincristine and cyclophosphamide are commonly used together in chemotherapy for neuroblastoma [34–36]. Second, they belong to two different categories of drugs: alkylating agents and plant alkaloids. As one crosslinks with DNA throughout the cell cycle and the other targets mitotic spindles during the M phase, they can conceivably complement each other. They are consistent with our mortality terms' mathematical form too. They do not only affect mitotic (dividing) cells, but also cells elsewhere in the cell cycle. Therefore, the chosen drugs are not necessarily compatible with the Norton-Simon hypothesis of treatment response [59], which argues that the rate of drug-induced mortality is proportional to the rate of cancer growth. Third, sufficient data were available for model calibration. Although we were not aware when we made the choice, we went on to find that VCR is less cytotoxic than CPM after model calibration. Our choice allowed us to discover therapeutic strategies involving a pair of differentially effective drugs.

After making this crucial choice, we parameterised different parts of the main equation separately with different experimental datasets. After parameterising one part of the equation, the relevant parametric values were retained for the remaining calibration steps. In most steps, a MatLab toolbox called lsqcurvefit, a non-linear least-squares solver, was used to fit parts of the equation to a relevant dataset.

The two clearance rates in the pharmacokinetic equations governing drug delivery were obtained directly from the literature. For a three-year-old child, 80 cm in height and 15 kg in weight, we found the raw clearance rates, 13.68 L h^{-1} m^{-2} [60] and 1.77 L h^{-1} m^{-2} [61], for VCR and CPM respectively. We used a formula to convert the patient's height and weight into the body surface area [62]. We found that the hypothetical patient's total body water is 58 % of the total body weight [63]. We multiplied each raw clearance rate by the ratio of the body surface area to the total body water, obtaining the clearance rates in the model: z_1 is 0.91 h^{-1} (VCR) and z_2 is 0.12 h^{-1} (CPM).

Tab.1 summarises the model parameters, including their symbols, numerical values, units, and physical meanings.

S3.1 Clonal growth rates in the absence of drugs

In the first instance, we simplified the model to consider the growth rate of one clone in the absence of drugs and other clones. We set $c_1 = 0$ and $c_2 = 0$ to reflect the drug-free environment, thus eliminating the death term D in the main equation governing clonal evolution, including $\phi_2(\tau)$. As the experiments providing the data lasted hours or days [64–66], we made the simplifying assumption that mutation had been negligible in the experiments, thus eliminating the mutation term M ($\mu = 0$). As phenotypic adaptation is irrelevant in a drug-free environment, we set $\tau = 0$ (no memory of chemotherapy), leading to the condition that $\phi_1(0) = 0$ (no phenotypic adaptation). The resulting minimal model is given by this equation,

$$\frac{dn_{i,j}}{dt} = \left(1 - \frac{n_{i,j}}{K}\right)\left(r_{i,j} n_{i,j}\right). \tag{S1}$$

We considered the minimal model, equation S1, for each clone separately. In each case, we used the same carrying capacity K. We chose a cell count consistent with the assumed maximum volume of a tumour in a three-year-old child: 10 cm^3.

First, for the sensitive clone, we used the average population doubling time of a continuous hyperdiploid human cell line, IMR-32, measured *in vitro* (chart 1 [64]). We found that $r_{0,0} = 8.5 \cdot 10^{-3}$ h^{-1}.

Second, we considered the mildly VCR-resistant clones. An experimental study generated VCR-resistant neuroblastoma cell lines by culturing neuroblastoma Be2c cells with VCR at gradually increasing concentrations [65]. The mildly VCR-resistant clone will be called VCR-10 henceforth because this cell line was resistant to a VCR concentration of 10 ng mL^{-1} in the experiment. A growth curve resulting from the experiment (Fig.1E [65]) allowed us to derive $r_{1,0} = 7.7 \cdot 10^{-3}$ h^{-1}, a fraction of $r_{0,0}$: reduced growth rate as a cost of genetically conferred resistance to VCR.

Third, we considered the strongly VCR-resistant clone. Its growth rate was derived from the observed doubling time of 25 hours [66]. The strongly VCR-resistant clone will be called VCR-20 henceforth because this cell line was resistant to a VCR concentration of 20 ng mL^{-1} in the experiment [66]. Equation (S1) was fitted to the *in vitro* data to calibrate $r_{2,0}$, leading to $r_{2,0} = 7.5 \cdot 10^{-3}$ h^{-1}.

In the absence of any published data, we could not calibrate the CPM-resistant clones' growth rates. The mildly and strongly CPM-resistant cells are called CPM-20 and CPM-32 cells respectively because they are resistant to doses of 20 and 32 mg kg^{-1} respectively. The clones with cross resistance presented the same challenge. We simply used the growth kinetics of the VCR-resistant clones to calibrate the corresponding growth rates for the remaining clones. In other words, the model assumes that phenotypic resistance to CPM has the same relationship with biomass production as VCR resistance. Moreover, CPM resistance is not affected by VCR resistance and *vice versa*. For example, this means that $r_{0,1} = r_{1,0}$ and $\frac{r_{1,1}}{r_{1,0}} = \frac{r_{1,0}}{r_{0,0}}$.

S3.2 Mutation probability

We made the simplifying assumption that the mutation probability μ takes the same value for all nine clones. As a range between 10^{-3} and 10^{-6} is reported [67] and we were designing a study of high-risk neuroblastoma (aggressive by definition), we decided that $\mu = 10^{-4}$ and forwent model fitting. Despite this simplistic choice, after calibrating the model, we managed to simulate a cancer cell population's recovery from rapid COJEC due to the emergence of resistant clones.

S3.3 Mortality rates

On the basis of the relevant experiments' time scales (hours or days) [65, 68–71], we made the simplifying assumption that the drug concentrations in the cultures had stayed constant throughout the experiments. On the same basis, we assumed that the cultured cells had neither adapted to the drugs phenotypically nor mutated. As genetically conferred resistance is drug-specific in this model, we only calibrated one mortality rate with respect to vincristine for each row in Fig.1 and one mortality rate with respect to cyclophosphamide for each column. For example, all three clones in the first row have the same mortality rate with respect to vincristine, while all three clones in the first column have the same mortality rate with respect to cyclophosphamide.

As explained in section S1, each mortality rate is represented by a non-linear function, $m_d^{i,j}(c_d) = \frac{m_{d,0}^{i,j} c_d}{1+\alpha_d c_d^{\beta_d}}$, so we needed to optimise the three parameters together for each pair of drug and clone. To achieve this task, we fitted equation (S2) to a relevant experimental dataset for each pair of drug and clone:

$$\frac{dn_{i,j}}{dt} = \left(1 - \frac{n_{i,j}}{K}\right)\left(r_{i,j} n_{i,j}\right) - \left(\frac{m_{d,0}^{i,j} c_d}{1 + \alpha_d c_d^{\beta_d}}\right) n_{i,j}. \tag{S2}$$

S3.3.1 Cytotoxic function of vincristine

We began our task by considering the mortality rates associated with VCR's cytotoxic function. In an experiment, sensitive neuroblasts were added to cultures with different concentrations of VCR and in each case, the extent of cell death was measured after four hours [68]. The measurements were used to produce a dose-response curve (Fig.4b [68]). To be consistent with this *in vitro* experiment, in this calibration step, we used a carrying capacity that reflects a well plate's capacity (not the one describing a child, reported in the main text): $K = 10^7$ cells. After fitting equation (S2) to the data, we found that $m_{1,0}^{0,0} = 41.8$ mg^{-1} m^2 h^{-1}, $\alpha = 1.122 \cdot 10^4$, and $\beta = 0.6704$. Armed with these parametric values, we reproduced the dose-response curve computationally, resulting in Fig.S1.

Calibration of VCR-10's and VCR-20's mortality rates posed a greater challenge due to data availability. For each of the two cell lines, as well as for sensitive cells, we only managed to find the drug concentration needed to inhibit half of the cells in a culture (IC_{50}) [65, 69]. At each level of resistance, multiple cell lines were used in the experimental study, so we took the average IC_{50} value for each level of resistance. As each average value of IC_{50} gave us one single datum only, we decided to keep the shape parameters ($\alpha_1 = 1.122 \cdot 10^4$ and $\beta_1 = 0.6704$) and calibrate $m_{1,0}^{1,0}$ and $m_{1,0}^{2,0}$ accordingly. For the sake of consistency, we recalibrated $m_{1,0}^{0,0}$ by keeping the shape parameters and reproducing the relevant IC_{50}. We found that

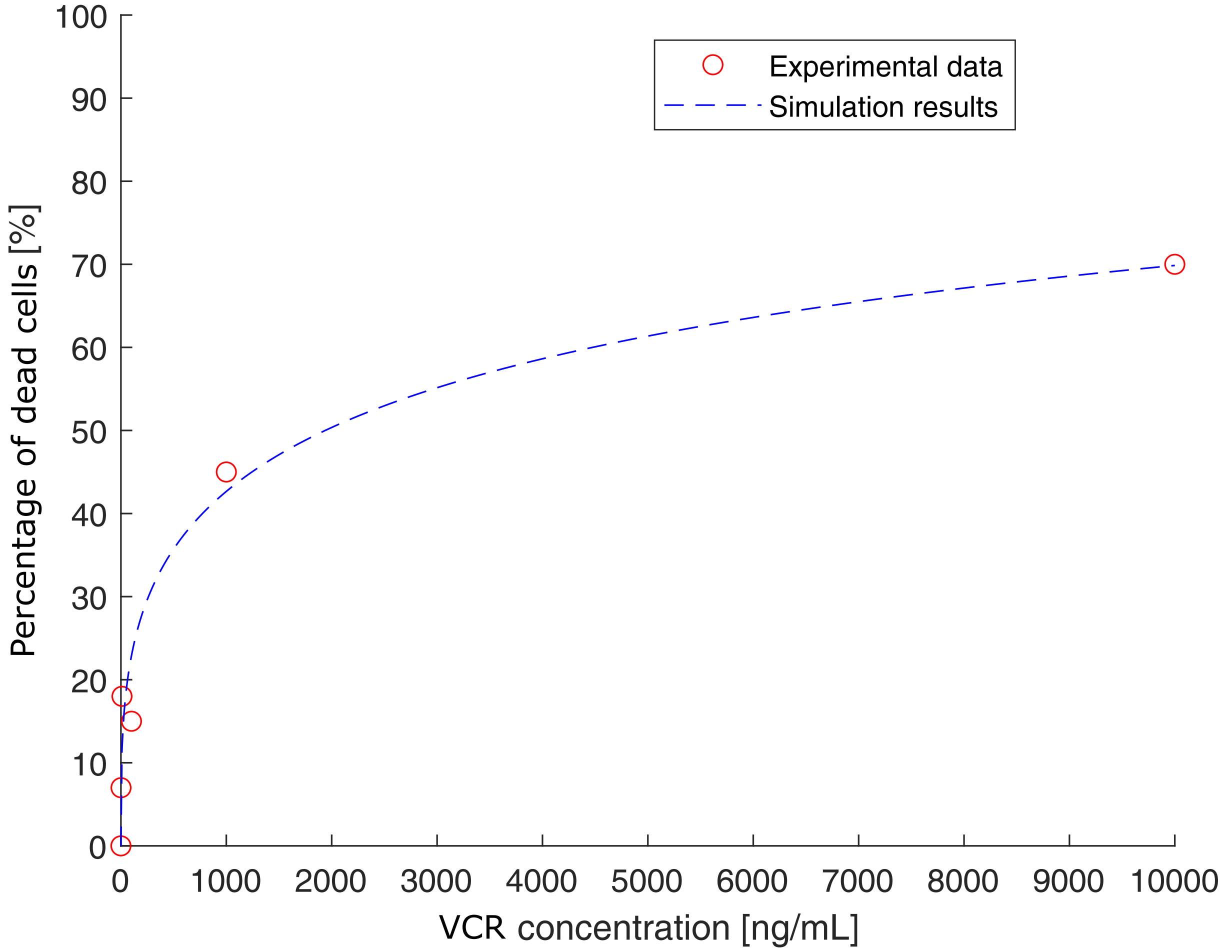

Figure S1: Saturation kinetics of sensitive neuroblasts responding to vincristine (VCR). In an experiment, sensitive neuroblasts were added to cultures with different concentrations of VCR and in each case, the extent of cell death was measured after four hours [68]. We used our calibrated model to reproduce the experiment. This figure reports, for each VCR concentration, the extent of cell death at equilibrium.

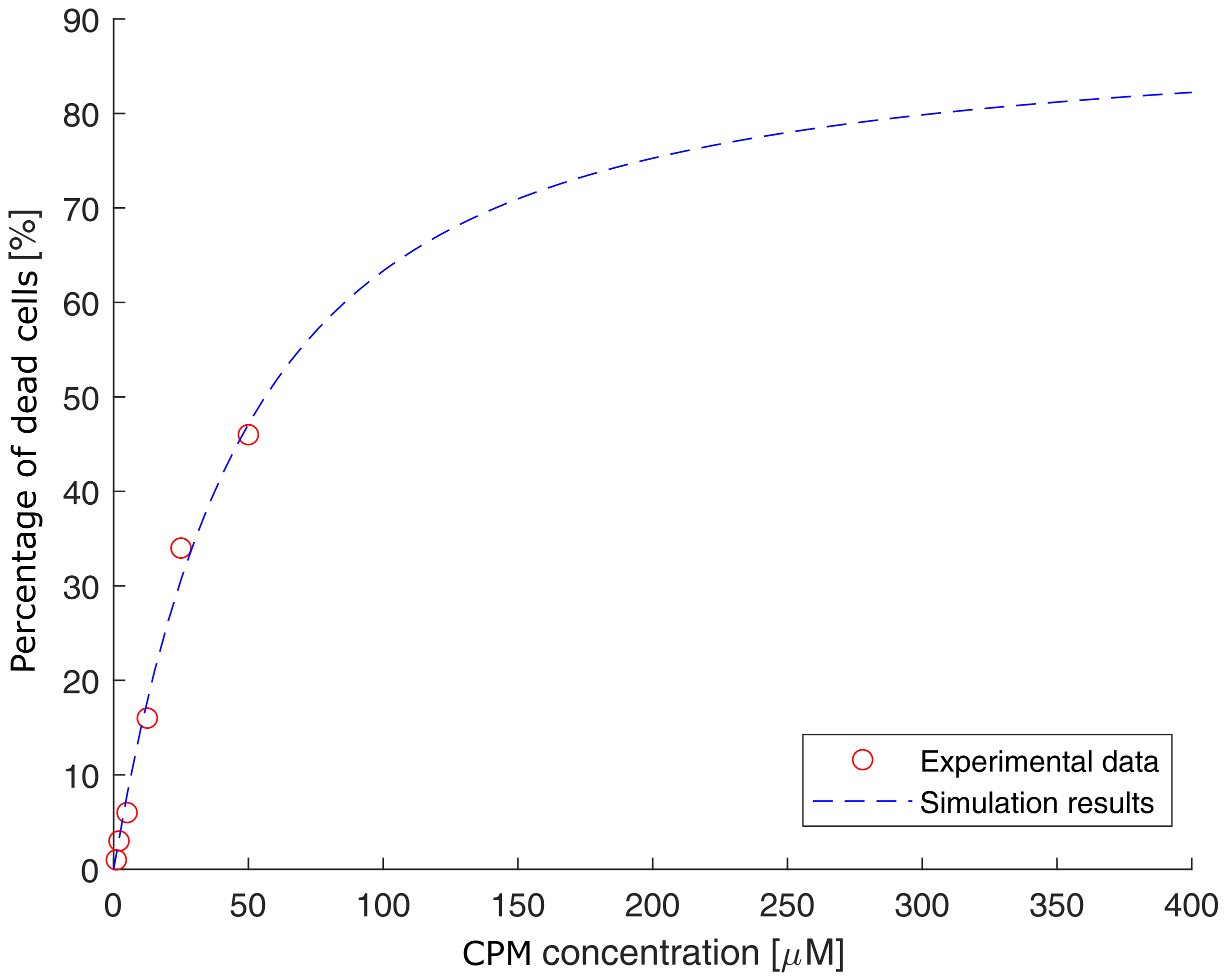

Figure S2: Saturation kinetics of sensitive neuroblasts responding to cyclophosphamide (CPM). In an experiment, CPM was used to treat neuroblastoma in a transgenic murine model for 24 hours [70]. Different CPM concentrations were used. We used our calibrated model to reproduce the experiment. This figure reports, for each CPM concentration, the extent of cell death at equilibrium.

$m_{1,0}^{0,0} = 40.4$ mg^{-1} m^2 h^{-1}, $m_{1,0}^{1,0} = 6.8$ mg^{-1} m^2 h^{-1}, and $m_{1,0}^{2,0} = 6$ mg^{-1} m^2 h^{-1}. It is noteworthy that the two estimates of $m_{1,0}^{0,0}$ (41.8 mg^{-1} m^2 h^{-1} and 40.4 mg^{-1} m^2 h^{-1}) are similar. We used the latter (40.4 mg^{-1} m^2 h^{-1}) for our dynamic simulations and optimisation studies.

S3.3.2 Cytotoxic function of cyclophosphamide

Then, we calibrated the mortality rates associated with CPM. In an experiment, CPM was used to treat neuroblastoma in a transgenic murine model for 24 hours [70], leading to a dose-response curve (Fig.5C [70]). We fitted equation (S2) to the data to find the parameters for the sensitive clone. For the carrying capacity (K), we chose a cell count consistent with the assumed maximum volume of a tumour in a three-year-old child: 10 cm^3. We found that $\alpha_2 = 2.9507 \cdot 10^{-5}$, $\beta_2 = 1$, and $m_{2,0}^{0,0} = 3.1474 \cdot 10^{-6}$ g^{-1} m^2 h^{-1}. We imposed that $\beta_2 = 1$ before fitting equation (S2) to the experimental data. This constraint led to a good match between the two, thus facilitating the calibration of α_2 and $m_{2,0}^{0,0}$. Armed with these parametric values, we reproduced the dose-response curve computationally, resulting in Fig.S2.

In the absence of suitable data, calibration of the CPM-resistant cells' mortality rates was more challenging. We first calibrated them with respect to experimental data about mice [71]. Then, we converted the calibrated values to human values.

In the experiment [71], Th-MYCN genetically engineered mice were exposed to multiple cycles of treatment with CPM. Changes in tumour size were tracked in 13 mice (Fig.1B, Fig.1C, and Fig.1G [71]). We

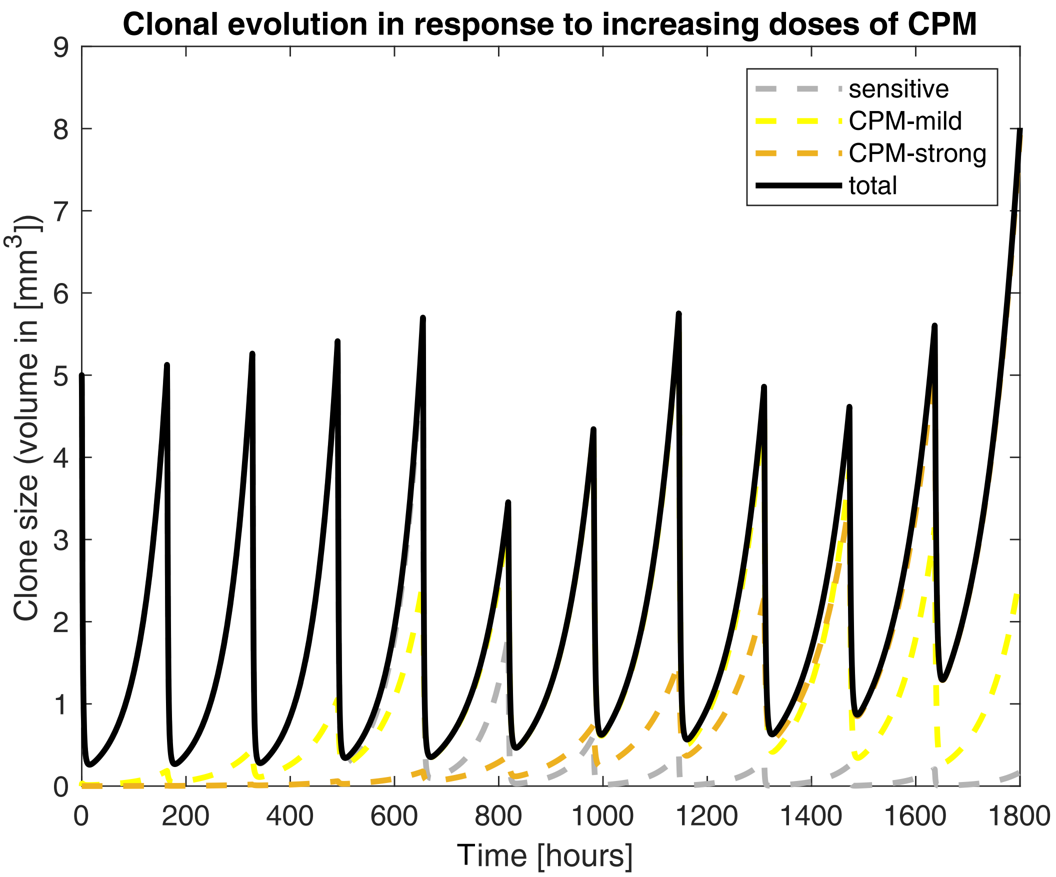

Figure S3: Clonal evolution driven by multiple cycles of treatment with cyclophosphamide (CPM). In an experiment [71], Th-MYCN genetically engineered mice were exposed to multiple CPM doses and changes in tumour size were tracked in 13 mice. We averaged the 13 drug schedules to obtain one representative schedule. In each of its first four cycles, 16 mg kg^{-1} of CPM was given. In the next three cycles, a higher dose (24 mg kg^{-1}) was used. In the last four cycles, the dose was increased further to 32 mg kg^{-1}. Each cycle lasted 164 hours. We used our calibrated model to simulate how CPM-resistant clones had emerged in the experiment. CPM was administered in the first hour of each cycle.

averaged the 13 drug schedules to design our own: 11 cycles (164 hours *per* cycle) of CPM treatment. In each of the first four cycles, 16 mg kg^{-1} of CPM was given. In the next three cycles, a higher dose (24 mg kg^{-1}) was used. In the last four cycles, the dose was increased further to 32 mg kg^{-1}.

Keeping the shape parameters for the sensitive clone ($\alpha_2 = 2.9507 \cdot 10^{-5}$ and $\beta_2 = 1$), we fitted equation (S2) and the pharmacokinetic equation for CPM ($\frac{dc_2}{dt} = \omega_2 - z_2 c_2$) to the data. For this calibration step and this calibration step only, we used a different value of z_2 (0.28 h^{-1} [72, 73]) because the experiment was performed on mice, not the three-year-old child our model describes. In the dynamic simulations associated with this calibration step, ω_2 was set to the experimental value in the first hour of each cycle and zero for the rest of the cycle, while ω_1 was set to zero throughout the simulations. To be consistent with the experiment [71], we imposed a hard constraint: the population became resistant to CPM after the 10th dose and before the 11th and last dose. Using the calibrated mortality rates, we reproduced the mice's average responses to the 11 doses of CPM: Fig.S3. Unlike the value of $m^{0,0}_{2,0}$ that we found in the last step ($3.1474 \cdot 10^{-6}$ g^{-1} m^2 h^{-1}), we found that $m^{0,0}_{2,0} = 33.9 * 3.1474 \cdot 10^{-6}$ g^{-1} m^2 h^{-1}. Furthermore, we found two relations: $m^{0,1}_{2,0} = 0.5 m^{0,0}_{2,0}$ and $m^{0,2}_{2,0} = 0.3 m^{0,0}_{2,0}$.

Finally, since the aim was to calibrate the model for a human child, not a mouse, we applied the two relations to the first value of $m^{0,0}_{2,0}$ ($3.1474 \cdot 10^{-6}$ g^{-1} m^2 h^{-1}). As a result, $m^{0,1}_{2,0} = 1.5737 \cdot 10^{-6}$ g^{-1} m^2 h^{-1} and $m^{0,2}_{2,0} = 9.4422 \cdot 10^{-7}$ g^{-1} m^2 h^{-1}.

S3.4 Phenotypic adaptation

As briefly mentioned in the section on our model's structure, cancer cells can phenotypically adapt to drugs in many ways, including epigenetic alterations. It is also known that these adaptive mechanisms, such as upregulating multidrug efflux pumps and EMT, are triggered by the cells' recent memory of their microenvironmental conditions, such as hypoxia and drugs [2, 74]. This lagging mechanism is parameterised by T_{max}, $\Phi_{1_{max}}$, and $\Phi_{2_{max}}$. Mathematically, $\phi_1(\tau)$ and $\phi_2(\tau)$ are linear functions that increase from $\Phi_{1_{min}} = \Phi_{2_{min}} = 0$ to $\Phi_{1_{max}}$ and $\Phi_{2_{max}}$ respectively as τ increases from $0 = T_{min}$ to T_{max}. In other words, $\phi_1(T_{min}) = \phi_1(0) = 0$, $\phi_2(T_{min}) = \phi_2(0) = 0$, $\phi_1(T_{max}) = \Phi_{1_{max}}$, and $\phi_2(T_{max}) = \Phi_{2_{max}}$. τ increases up to T_{max} when the tumour is under drug stress (the constraint $c_1 + c_2 > 0.01$ is met); otherwise, τ decreases up to 0 (T_{min}) when the tumour is not under drug stress (the constraint $c_1 + c_2 > 0.01$ is violated).

In this part of the calibration process, we turned to one of the experimental studies used to calibrate the clonal growth rates in the absence of drugs. The experimental study generated VCR-resistant neuroblastoma cell lines by culturing neuroblastoma Be2c cells with VCR at gradually increasing concentrations [65]. The abundances of sensitive and VCR-20 cells cultured with and without VCR were measured (Fig.2B [65]). First, the green bar pertains to untreated sensitive cells, while the dark green bar pertains to sensitive cells treated with 20 ng mL^{-1} of VCR for 48 hours. Second, the grey bar corresponds to VCR-20 cells kept in a medium with VCR. Third, although both the blue and dark blue bars describe VCR-20 cells cultured for three weeks in a VCR-free medium, the cells described by the latter were treated with 20 ng mL^{-1} of VCR for another 48 hours. To be consistent with this *in vitro* experiment, in this calibration step, we used a carrying capacity that reflects a well plate's capacity (not the one describing a child, reported in the main text): $K = 10^7$ cells.

The first parameters, $\Phi_{1_{min}}$, $\Phi_{2_{min}}$, T_{min}, T_{max}, and the function type, were calibrated in a straightforward manner. We fixed $\Phi_{1_{min}} = \Phi_{2_{min}} = 0$, corresponding to no phenotypic effects. $T_{min} = 0$ and $T_{max} = 10$ days since the cancer cells were cultured for three weeks in drug-free media [65]: an indication that the plastic response can fully activate or deactivate and reach the regimen in 21 days. Finally, we decided to use the most common function type for ϕ_1 and ϕ_2, i.e., linear functions.

On the basis of the time scale (hours to days) [65], we made the assumption that the cultured cells had not mutated. Mathematically, this assumption allowed us to reduce the main equation to the following,

$$\frac{dn_{i,j}}{dt} = \left(1 - \frac{n_{i,j}}{K}\right)\left(\frac{r_{i,j}n_{i,j}}{1+\phi_1(\tau)}\right) - \left(\frac{m_{d,0}^{i,j}c_d}{1+\alpha_d c_d^{\beta_d}}\right)\left(\frac{n_{i,j}}{1+\phi_2(\tau)}\right). \tag{S3}$$

Drug resistance achieved via phenotypic adaptation (such as ATP-consuming efflux pumps) comes at the expense of other biological processes (such as growth) and informed by the literature, we made the assumption that maximum adaptation or maximum ATP consumption comes at the expense of a 50 % reduction in growth [75, 76]. Since the assumption means that $\left(1 - \frac{n_{i,j}}{K}\right)\left(\frac{r_{i,j}n_{i,j}}{1+\Phi_{1_{max}}}\right)$ is half of $\left(1 - \frac{n_{i,j}}{K}\right)\left(r_{i,j}n_{i,j}\right)$, it follows from the assumption that $\Phi_{1_{max}} = 1$.

With $\Phi_{1_{max}} = \phi_1(T_{max}) = 1$, we fitted equation (S3) to the data relating to untreated sensitive cells and sensitive cells treated with 20 ng mL^{-1} of VCR for 48 hours (green and dark green bars in Fig.2B [65]). We arrived at the conclusion that $\Phi_{2_{max}} = \phi_2(T_{max}) = 2$, rounded off from 1.868. Using the calibrated parameters, we reproduced this part of the experiment computationally: left panel of Fig.S4. In fact, Fig.S4 suggests that we successfully reproduced all three parts of the experiment: the effect of VCR on sensitive neuroblastoma (the green and dark green bars), the phenotypic adaptation to VCR of VCR-20 cells (the grey bar), and the effects of VCR on VCR-20 cells without phenotypic adaptation (the blue and dark blue bars).

S4 Model Validation

After calibration, we performed three tests to validate our calibrated model.

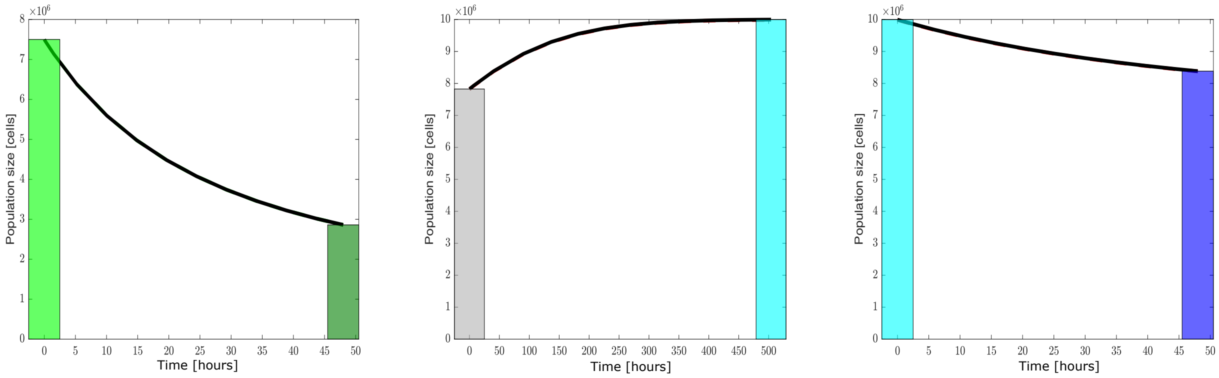

Figure S4: Simulated population sizes in cultures with different neuroblastoma cell lines with and without vincristine (VCR). The experimental study generated VCR-resistant neuroblastoma cell lines by culturing neuroblastoma Be2c cells with VCR at gradually increasing concentrations [65]. The growth rates of sensitive and VCR-20 (strongly VCR-resistant) cells cultured with and without VCR were measured. First, the green bar pertains to untreated sensitive cells, while the dark green bar pertains to sensitive cells treated with 20 ng mL^{-1} of VCR for 48 hours. Second, the grey bar is about VCR-20 cells kept in a medium with VCR. Third, although both the blue and dark blue bars describe VCR-20 cells cultured for three weeks in a VCR-free medium, the cells described by the latter were treated with 20 ng mL^{-1} of VCR for another 48 hours. In each panel, the black line represents the number of cells.

S4.1 Dynamics of VCR-20 cells in different scenarios

As already explained alongside the calibration of phenotypic adaptation (T_{max}, $\Phi_{1_{max}}$, and $\Phi_{1_{max}}$), we successfully simulated the experimentally observed dynamics of VCR-20 cells in different scenarios [65]. The right panel of Fig.S4 shows that the simulated VCR-20 population declined in size by around 15 % because this population's phenotypic adaptation was inactive during the simulation, in agreement with the *in vitro* experiment [65].

S4.2 Consistent emergence of drug-resistant clones

We assessed its ability to demonstrate basic, intuitive biological phenomena. As discussed in [77], cancer cells usually develop resistance to a drug due to the many mechanisms available to them in the human genome. Starting with different initial clonal compositions, we used the calibrated model to simulate the population dynamics in the presence of VCR alone, CPM alone, and both drugs. In each case, as the simulation continued, the clones resistant to the administered drug or drugs expanded logistically, outcompeted the other clones, and dominated the tumour population.

S4.3 Demonstration of multidrug resistance

We tested our calibrated model's ability to demonstrate multidrug resistance. We did so by reproducing experimental observations of various CPM-resistant cell lines responding to VCR at different concentrations (Supplementary Fig.6G [71]). The simulation results, summarised in Fig.S5, indicate that on average, our model adheres to the experimental observations.

S5 Optimising Chemotherapy Schedules

Rapid COJEC is a chemotherapy regimen used in the induction phase of the multi-modal therapy for high-risk neuroblastoma [78, 79]. It uses fixed doses of chemotherapeutic agents in eight two-week cycles. It is a one-size-fits-all regimen in the sense that the maximum tolerated dose (MTD) is used for all patients.

Using the final population size (i.e., the sum of the nine clones two weeks after the beginning of the last cycle) as the objective function to be minimised, we solved a series of optimisation problems to identify

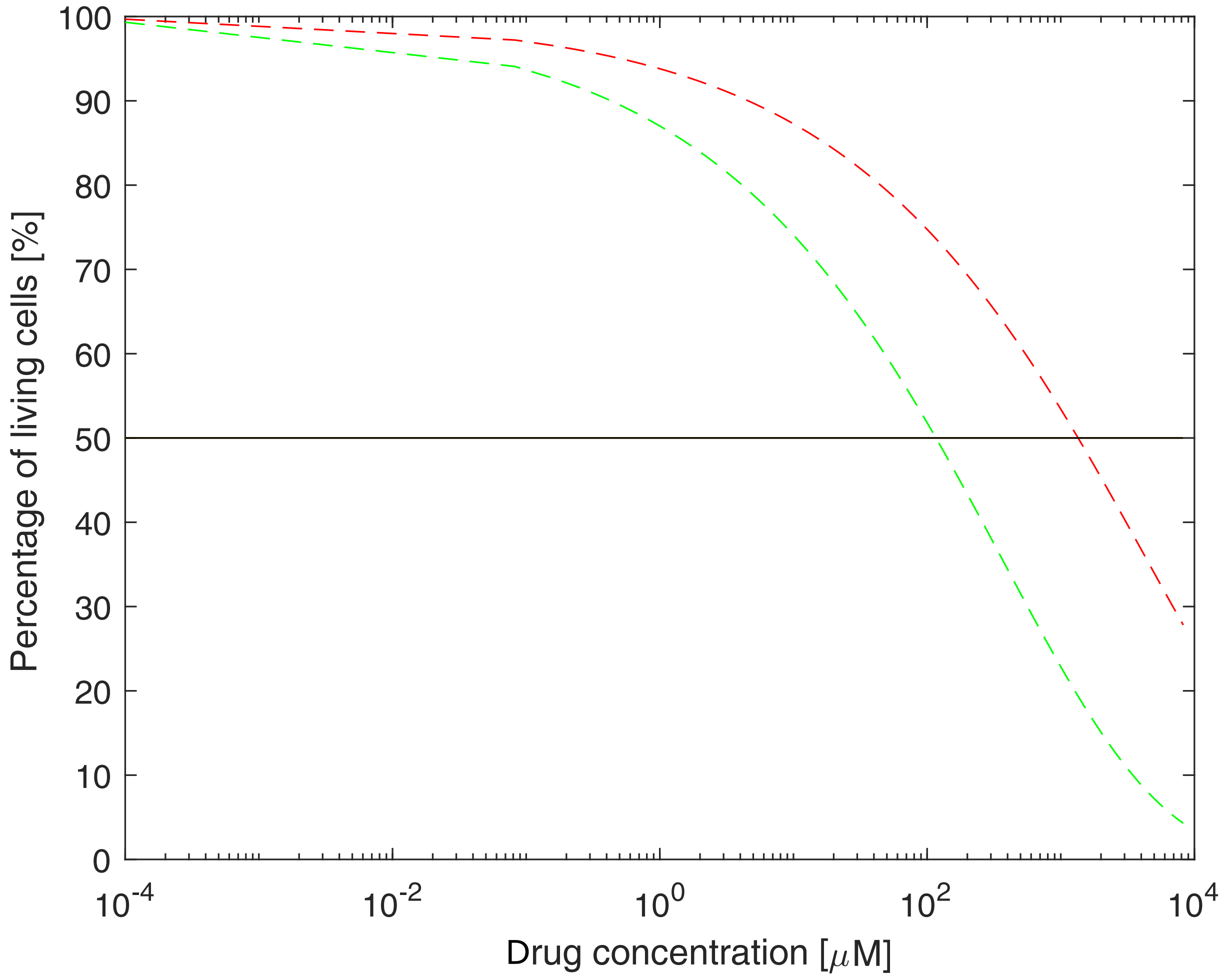

Figure S5: Demonstration of multidrug resistance in simulations. In an experiment, various cyclophosphamide (CPM)-resistant cell lines were treated with vincristine (VCR) at different concentrations [71]. We used the calibrated model to reproduce the study. This figure reports the equilibria reached by sensitive (green line) and CPM-resistant (red line) cells in simulations at different VCR concentrations.

ways of improving rapid COJEC. While maintaining the fundamental unit (a two-week cycle), we experimented with the dosage: the number of cycles and the doses in each cycle. We limited our solution space to 12 or fewer cycles due to concerns about toxicity, but the exact choice of considering up to 50% more cycles than the standard protocol was made *ad hoc*. In each cycle, we limited the dose of VCR to its MTD (2 mg m^{-2}) [80] and did the same for CPM (2 g m^{-2}) [81]. In each cycle, the VCR dose was equally administered in the first 48 hours [80], while the CPM dose was administered in the first 56 hours [81]. According to the sources [80,81], VCR is administered intravenously as a solution, while CPM is administered as powder, hence the different periods of administration in our studies. Depending on the dose units we found in the literature, we adjusted the units of c_d and $m_{d,0}^{i,j}$ to ensure meaningful quantities were used during calibration, the optimisation studies, and the dynamic simulations. Therefore, in each optimisation problem, we varied the number of cycles from one to 12 and in each case, we optimised up to 24 control variables: two doses in each cycle.

We solved each optimisation problem by combining a global search method with a local approach. As we had decided to limit our study to 12 or fewer cycles, we searched 12 solution spaces (two to 24 dimensions) globally with a genetic algorithm (GA) [82] before passing the proposed solutions to our local method. The local method, MatLab's fmincon function, was used to look for an even better solution in the local vicinity of each GA proposed solution. GAs belong to the family of algorithms for global optimisation called evolutionary computation. A GA looks for the optimal solution that maximises a fitness function by passing potential solutions through the evolutionarily inspired processes of selection, crossover, and mutation iteratively.

In the global part of each problem, we set the fitness function of our GA to the inverse of our objective function. Recall that our objective function is the sum of the nine cell counts ($n_{i,j}$) at the end of the last treatment cycle. Then, we started with 100 random solutions (100 ordered lists of 24 numbers), or 100 chromosomes with 24 genes apiece within the context of a GA. We passed these chromosomes through selection, crossover, and mutation 1000 times or generations. In each generation, the following steps were carried out.

- The 10 fittest chromosomes entered the next generation without modification.
- During crossover, the existing chromosomes were sampled stochastically with replacement according to their relative fitness levels. Two selected chromosomes were paired up and a crossover point was randomly selected. The genes before this point were merged in a random convex combination (sum of the two weights is one) and this combination was passed on to two daughter chromosomes, while the genes after this point were passed on without change from one parent to one daughter. Then, each gene on each daughter chromosome was mutated (assigned a random valid value) with a probability of 5 %. This step created 50 daughter chromosomes.
- 40 more chromosomes were randomly generated to make up the next generation. These 'immigrants' allowed us to explore the solution space more widely at the expense of improving the fit chromosomes of the current generation.

For each optimisation problem, we found the optimal chemotherapy schedule for a different initial tumour composition. First, we considered a virtual tumour initially comprising fully sensitive cells only. Then, for each of the following seven scenarios, we considered five initial tumour compositions: 5, 10, 15, 20, and 25 % of the cells were resistant initially.

- All the resistant cells were set to be VCR-10 cells initially.
- All the resistant cells were set to be VCR-20 cells initially.
- All the resistant cells were set to be CPM-20 cells initially.
- All the resistant cells were set to be CPM-32 cells initially.
- Half of the resistant cells were set to be VCR-10 cells and the other half to CPM-20 cells initially.
- Half of the resistant cells were set to be VCR-20 cells and the other half to CPM-32 cells initially.
- The resistant cells were divided between the four resistant types: a third were VCR-10, another third were CPM-20, a sixth were VCR-20, and the remaining sixth were CPM-32.

Therefore, we solved 36 optimisation problems. When we tackled each problem, we optimised the doses for a fixed number of cycles before lengthening the schedule, going from one to 12 cycles. In total, we implemented our GA-fmincon method 432 times on an Intel Xeon(R) Processor E5-2650 (2.00 GHz). For a particular initial tumour composition, with the number of treatment cycles fixed at eight, the algorithm took around 32800 seconds (almost nine hours) to finish. Together, Fig.2 presents the optimal chemotherapy schedules we found by solving the optimisation problems.

References

[1] L J Goldstein, A T Fojo, K Ueda, W Crist, A Green, G BrodeurI Pastan, and M M Gottesman. Expression of the multidrug resistance, mdr1, gene in neuroblastomas. *Journal of Clinical Oncology*, 2009.

[2] Adam M Feist and Bernhard O Palsson. The biomass objective function. *Current opinion in microbiology*, 13(3):344–349, 2010.

[3] Ranju Ralhan and Jatinder Kaur. Alkylating agents and cancer therapy. *Expert Opinion on Therapeutic Patents*, 17(9):1061–1075, 2007.

[4] Shaloam Dasari and Paul Bernard Tchounwou. Cisplatin in cancer therapy: molecular mechanisms of action. *European journal of pharmacology*, 740:364–378, 2014.

[5] Dong-Wan Kim, Yo-Han Jo, Jee Hyun Kim, Hong-Gyun Wu, Chae Seo Rhee, Chol Hee Lee, Tae-You Kim, Dae Seog Heo, Yung-Jue Bang, and Noe Kyeong Kim. Neoadjuvant etoposide, ifosfamide, and cisplatin for the treatment of olfactory neuroblastoma. *Cancer: Interdisciplinary International Journal of the American Cancer Society*, 101(10):2257–2260, 2004.

[6] F Ann Hayes, Alexander A Green, James Casper, Joann Cornet, and William E Evans. Clinical evaluation of sequentially scheduled cisplatin and vm26 in neuroblastoma: response and toxicity. *Cancer*, 48(8):1715–1718, 1981.

[7] T Philip, R Ghalie, R Pinkerton, JM Zucker, JL Bernard, G Leverger, and O Hartmann. A phase ii study of high-dose cisplatin and vp-16 in neuroblastoma: a report from the société française d'oncologie pédiatrique. *Journal of Clinical Oncology*, 5(6):941–950, 1987.

[8] Olga Piskareva, Harry Harvey, John Nolan, Ross Conlon, Leah Alcock, Patrick Buckley, Paul Dowling, Michael Henry, Finbarr O'Sullivan, Isabella Bray, et al. The development of cisplatin resistance in neuroblastoma is accompanied by epithelial to mesenchymal transition in vitro. *Cancer letters*, 364(2):142–155, 2015.

[9] Miguel Angel Merlos Rodrigo, Hana Buchtelova, Ana Maria Jimenez Jimenez, Pavlina Adam, Petr Babula, Zbynek Heger, and Vojtech Adam. Transcriptomic landscape of cisplatin-resistant neuroblastoma cells. *Cells*, 8(3):235, 2019.

[10] Anthony J Di Pasqua, Jerry Goodisman, and James C Dabrowiak. Understanding how the platinum anticancer drug carboplatin works: From the bottle to the cell. *Inorganica Chimica Acta*, 389:29–35, 2012.

[11] Hervé Rubie, C Coze, D Plantaz, C Munzer, AS Defachelles, C Bergeron, C Thomas, P Chastagner, D Valteau-Couanet, J Michon, et al. Localised and unresectable neuroblastoma in infants: excellent outcome with low-dose primary chemotherapy. *British journal of cancer*, 89(9):1605–1609, 2003.

[12] H Rubie, J Michon, D Plantaz, MC Peyroulet, C Coze, D Frappaz, P Chastagner, MC Baranzelli, F Mechinaud, P Boutard, et al. Unresectable localized neuroblastoma: improved survival after primary chemotherapy including carboplatin-etoposide. *British journal of cancer*, 77(12):2310–2317, 1998.

[13] David J Stewart. Mechanisms of resistance to cisplatin and carboplatin. *Critical reviews in oncology/hematology*, 63(1):12–31, 2007.

[14] Timothy C Johnstone, Kogularamanan Suntharalingam, and Stephen J Lippard. The next generation of platinum drugs: targeted pt (ii) agents, nanoparticle delivery, and pt (iv) prodrugs. *Chemical reviews*, 116(5):3436–3486, 2016.

[15] Nci drug dictionary, iproplatin. https://www.cancer.gov/publications/dictionaries/cancer-drug/def/iproplatin. Accessed: 2022-06-30.

[16] Mari H Ogino and Prasanna Tadi. Cyclophosphamide. 2020.

[17] Borje Andersson and David Murray. *Clinically relevant resistance in cancer chemotherapy*, volume 112. Springer Science & Business Media, 2002.

[18] Aengus S O'Marcaigh and Donna L Betcher. Busulfan. *Journal of Pediatric Oncology Nursing*, 13(3):150–152, 1996.

[19] Virginia Probin, Yong Wang, and Daohong Zhou. Busulfan-induced senescence is dependent on ros production upstream of the mapk pathway. *Free Radical Biology and Medicine*, 42(12):1858–1865, 2007.

[20] S Proust-Houdemont, C Pasqualini, P Blanchard, C Dufour, E Benhamou, G Goma, M Semeraro, MA Raquin, O Hartmann, and D Valteau-Couanet. Busulfan–melphalan in high-risk neuroblastoma: the 30-year experience of a single institution. *Bone marrow transplantation*, 51(8):1076–1081, 2016.

[21] Benigno C Valdez, David Murray, Latha Ramdas, Marcos De Lima, Roy Jones, Steven Kornblau, Daniel Betancourt, Yang Li, Richard E Champlin, and Borje S Andersson. Altered gene expression in busulfan-resistant human myeloid leukemia. *Leukemia research*, 32(11):1684–1697, 2008.

[22] MJ Van Maanen, CJM Smeets, and JH Beijnen. Chemistry, pharmacology and pharmacokinetics of n, n, n-triethylenethiophosphoramide (thiotepa). *Cancer treatment reviews*, 26(4):257–268, 2000.

[23] C Pasqualini, C Dufour, G Goma, MA Raquin, V Lapierre, and D Valteau-Couanet. Tandem high-dose chemotherapy with thiotepa and busulfan–melphalan and autologous stem cell transplantation in very high-risk neuroblastoma patients. *Bone Marrow Transplantation*, 51(2):227–231, 2016.

[24] Shu-Ting Pan, Zhi-Ling Li, Zhi-Xu He, Jia-Xuan Qiu, and Shu-Feng Zhou. Molecular mechanisms for tumour resistance to chemotherapy. *Clinical and Experimental Pharmacology and Physiology*, 43(8):723–737, 2016.

[25] Mounika Gangireddy and Vinod Nookala. Ifosfamide. In *StatPearls [Internet]*. StatPearls Publishing, 2021.

[26] Susan M Ludeman and Michael P Gamcsik. Mechanisms of resistance against cyclophosphamide and ifosfamide: can they be overcome without sacrificing selectivity? *Clinically Relevant Resistance in Cancer Chemotherapy*, pages 177–197, 2002.

[27] Rajesh Thirumaran, George C Prendergast, and Paul B Gilman. Cytotoxic chemotherapy in clinical treatment of cancer. In *Cancer immunotherapy*, pages 101–116. Elsevier, 2007.

[28] Ruth Ladenstein, Ulrike Pötschger, Andrew DJ Pearson, Penelope Brock, Roberto Luksch, Victoria Castel, Isaac Yaniv, Vassilios Papadakis, Geneviève Laureys, Josef Malis, et al. Busulfan and melphalan versus carboplatin, etoposide, and melphalan as high-dose chemotherapy for high-risk neuroblastoma (hr-nbl1/siopen): an international, randomised, multi-arm, open-label, phase 3 trial. *The lancet oncology*, 18(4):500–514, 2017.

[29] Qing Chen, Pieter C Van der Sluis, David Boulware, Lori A Hazlehurst, and William S Dalton. The fa/brca pathway is involved in melphalan-induced dna interstrand cross-link repair and accounts for melphalan resistance in multiple myeloma cells. *Blood*, 106(2):698–705, 2005.

[30] JR Wesolowski, P Rajdev, and SK Mukherji. Temozolomide (temodar). *American journal of neuroradiology*, 31(8):1383–1384, 2010.

[31] Guo-zhong Yi, Guanglong Huang, Manlan Guo, Xi'an Zhang, Hai Wang, Shengze Deng, Yaomin Li, Wei Xiang, Ziyang Chen, Jun Pan, et al. Acquired temozolomide resistance in mgmt-deficient glioblastoma cells is associated with regulation of dna repair by dhc2. *Brain*, 142(8):2352–2366, 2019.

[32] Thomas Efferth and Franz Oesch. Repurposing of plant alkaloids for cancer therapy: Pharmacology and toxicology. In *Seminars in Cancer Biology*, volume 68, pages 143–163. Elsevier, 2021.

[33] Yue Tu, Shixiang Cheng, Sai Zhang, Hongtao Sun, and Zhongwei Xu. Vincristine induces cell cycle arrest and apoptosis in sh-sy5y human neuroblastoma cells. *International journal of molecular medicine*, 31(1):113–119, 2013.

[34] Steven G DuBois, Margaret E Macy, and Tara O Henderson. High-risk and relapsed neuroblastoma: Toward more cures and better outcomes. *American Society of Clinical Oncology Educational Book*, 42:1–13, 2022.

[35] Brian H Kushner, Kim Kramer, Shakeel Modak, Li-Xuan Qin, and Nai-Kong V Cheung. Differential impact of high-dose cyclophosphamide, topotecan, and vincristine in clinical subsets of patients with chemoresistant neuroblastoma. *Cancer*, 116(12):3054–3060, 2010.

[36] Audrey E Evans, Ruth M Heyn, William A Newton, and Sanford L Leikin. Vincristine sulfate and cyclophosphamide for children with metastatic neuroblastoma. *JAMA*, 207(7):1325–1327, 1969.

[37] Zhongjie Xu and Lirong Zhang. Brca1 expression serves a role in vincristine resistance in colon cancer cells. *Oncology Letters*, 14(1):345–348, 2017.

[38] Masanobu Tsubaki, Tomoya Takeda, Naoki Ogawa, Kotaro Sakamoto, Hirotaka Shimaoka, Arisa Fujita, Tatsuki Itoh, Motohiro Imano, Toshihiko Ishizaka, Takao Satou, et al. Overexpression of survivin via activation of erk1/2, akt, and nf-κb plays a central role in vincristine resistance in multiple myeloma cells. *Leukemia research*, 39(4):445–452, 2015.

[39] Yuzhen Xu and Liyan Qiu. Nonspecifically enhanced therapeutic effects of vincristine on multidrug-resistant cancers when coencapsulated with quinine in liposomes. *International journal of nanomedicine*, 10:4225, 2015.

[40] JJM Holthuis. Etoposide and teniposide. *Pharmaceutisch Weekblad*, 10(3):101–116, 1988.

[41] Kenneth R Hande. Topoisomerase ii inhibitors. *Update on cancer therapeutics*, 3(1):13–26, 2008.

[42] Ram N Ganapathi and Mahrukh K Ganapathi. Mechanisms regulating resistance to inhibitors of topoisomerase ii. *Frontiers in pharmacology*, 4:89, 2013.

[43] Z Ping Lin, Yoonkyung C Boller, Suad M Amer, Rosalind L Russell, Karen A Pacelli, Steven R Patierno, and Katherine A Kennedy. Prevention of brefeldin a-induced resistance to teniposide by the proteasome inhibitor mg-132: involvement of nf-κb activation in drug resistance. *Cancer Research*, 58(14):3059–3065, 1998.

[44] Yves Pommier. Topoisomerase i inhibitors: camptothecins and beyond. *Nature Reviews Cancer*, 6(10):789–802, 2006.

[45] Shantanu Banerji and Marek Los. Important differences between topoisomerase-i and-ii targeting agents. *Cancer biology & therapy*, 5(8):965–966, 2006.

[46] Zeshaan A Rasheed and Eric H Rubin. Mechanisms of resistance to topoisomerase i-targeting drugs. *Oncogene*, 22(47):7296–7304, 2003.

[47] Yuan Gao, Qingyao Shang, Wenyu Li, Wenxuan Guo, Alexander Stojadinovic, Ciaran Mannion, Yan-gao Man, and Tingtao Chen. Antibiotics for cancer treatment: A double-edged sword. *Journal of Cancer*, 11(17):5135, 2020.

[48] Oktay Tacar, Pornsak Sriamornsak, and Crispin R Dass. Doxorubicin: an update on anticancer molecular action, toxicity and novel drug delivery systems. *Journal of pharmacy and pharmacology*, 65(2):157–170, 2013.

[49] Kanu Chatterjee, Jianqing Zhang, Norman Honbo, and Joel S Karliner. Doxorubicin cardiomyopathy. *Cardiology*, 115(2):155–162, 2010.

[50] Claudia Christowitz, Tanja Davis, Ashwin Isaacs, Gustav Van Niekerk, Suzel Hattingh, and Anna-Mart Engelbrecht. Mechanisms of doxorubicin-induced drug resistance and drug resistant tumour growth in a murine breast tumour model. *BMC cancer*, 19(1):1–10, 2019.

[51] Caroline F Thorn, Connie Oshiro, Sharon Marsh, Tina Hernandez-Boussard, Howard McLeod, Teri E Klein, and Russ B Altman. Doxorubicin pathways: pharmacodynamics and adverse effects. *Pharmacogenetics and genomics*, 21(7):440, 2011.

[52] Douglas Ormrod, Kristin Holm, Karen Goa, and Caroline Spencer. Epirubicin. *Drugs & aging*, 15(5):389–416, 1999.

[53] KK Matthay, JM Maris, G Schleiermacher, A Nakagawara, CL Mackall, L Diller, and WA Weiss. Neuroblastoma. nature reviews. disease primers 2, 16078, 2016.

[54] Katherine K Matthay, Judith G Villablanca, Robert C Seeger, Daniel O Stram, Richard E Harris, Norma K Ramsay, Patrick Swift, Hiroyuki Shimada, C Thomas Black, Garrett M Brodeur, et al. Treatment of high-risk neuroblastoma with intensive chemotherapy, radiotherapy, autologous bone marrow transplantation, and 13-cis-retinoic acid. *New England Journal of Medicine*, 341(16):1165–1173, 1999.

[55] C Patrick Reynolds, Katherine K Matthay, Judith G Villablanca, and Barry J Maurer. Retinoid therapy of high-risk neuroblastoma. *Cancer letters*, 197(1-2):185–192, 2003.

[56] Michelle E Keyel and C Patrick Reynolds. Spotlight on dinutuximab in the treatment of high-risk neuroblastoma: development and place in therapy. *Biologics: targets & therapy*, 13:1, 2019.

[57] Julie Voeller and Paul M Sondel. Advances in anti-gd2 immunotherapy for treatment of high-risk neuroblastoma. *Journal of pediatric hematology/oncology*, 41(3):163, 2019.

[58] Salvatore Raieli, Daniele Di Renzo, Silvia Lampis, Camilla Amadesi, Luca Montemurro, Andrea Pession, Patrizia Hrelia, Matthias Fischer, and Roberto Tonelli. Mycn drives a tumor immunosuppressive environment which impacts survival in neuroblastoma. *Frontiers in oncology*, 11:625207, 2021.

[59] Tiffany A. Traina and Larry Norton. *Norton-Simon Hypothesis*, pages 2557–2559. Springer Berlin Heidelberg, Berlin, Heidelberg, 2011.

[60] Ellis Groninger, Tiny Meeuwsen de Boar, Pauline Koopmans, Donald Uges, Wim Sluiter, Anjo Veerman, Willem Kamps, and Siebold de Graaf. Pharmacokinetics of vincristine monotherapy in childhood acute lymphoblastic leukemia. *Pediatric Research*, 2002.

[61] Jeannine S. McCune, David H. Salinger, Paolo Vicini, Celeste Oglesby, David K. Blough, and Julie R. Park. Population pharmacokinetics of cyclophosphamide and metabolites in children with neuroblastoma: a report from the children's oncology group. *Journal of Clinical Pharmacology*, 2009.

[62] Janusz Feber and Hana Krásničanová. Measures of body surface area in children. *Handbook of Anthropometry: Physical Measures of Human Form in Health and Disease*, pages 1249–1256, 2012.

[63] Bent J. Friis-Hansen, Malco Holiday, Thomas Stapleton, and William M. Wallace. Total body water in children. *Pediatrics*, 7(3):321–327, 1951.

[64] Joseph J. Tumilowicz, Warren W. Nichols, Jolanta J. Cholon, and Arthur E. Greene. Definition of a continuous human cell line derived from neuroblastoma. *Cancer Research*, 30(08):2110–2118, 1970.

[65] Mohamed Jemaà, Wondossen Sime, Yasmin Abassi, Vito Alessandro Lasorsa, Julie Bonne Køhler, Martin Michaelis, Jindrich Cinatl, Mario Capasso, and Ramin Massoumi. Gene expression signature of acquired chemoresistance in neuroblastoma cells. *International Journal of Molecular Sciences 21(18),6811, 1-153*, 21(18):6811, 1–15, 2020.

[66] Rouslana Kotchetkov, Jaroslavc Cinatl, Romanb Blaheta, Jens-Uweb Vogel, Janad Karaskova, Jeremyd Squire, Pablo Hernáize Driever, Thomasa Klingebiel, and Jindrich Cinatl Jr. Development of resistance to vincristine and doxorubicin in neuroblastoma alters malignant properties and induces additional karyotype changes: a preclinical model. *International Journal of Cancer.*

[67] Petera Duesberg, Reinhard Stindl, and Rüediger Hehlmann. Explaining the high mutation rates of cancer cells to drug and multidrug resistance by chromosome reassortments that are catalyzed by aneuploidy. *Proceedings of the National Academy of Sciences of the United States of America*, 97(26):14295–14300, 2000.

[68] Y. Zaizen, A. Nakagawara, and K Ikeda. Patterns of destruction of mouse neuroblastoma cells by extracellular hydrogen peroxide formed by 6-hydroxydopamine and ascorbate. *Journal of Cancer Research and Clinical Oncology*, 111(2):93–97, 1986.

[69] Nadine Löschmann, Martin Michaelis, Florian Rothweiler, Richard Zehner, Jaroslav Cinatl, Yvonne Voges, Mohsen Sharifi, Kristoffer Riecken, Jochen Meyer, Andreas von Deimling, Iduna Fichtner, Taravat Ghafourian, Frank Westermann, and Jr Jindrich Cinatl. Testing of sns-032 in a panel of human neuroblastoma cell lines with acquired resistance to a broad range of drugs. *Translational Oncology*, 6(6):685–696, 2013.

[70] Louis Chesler, David D. Goldenberg, Rodney Collins, Matt Grimmer, Grace E. Kim, Tarik Tihan, Kim Nguyen, Slava Yakovenko, Katherine K. Matthay, and William A. Weiss. Chemotherapy-induced apoptosis in a transgenic model of neuroblastoma proceeds through p53 induction. *Neoplasia*, 10(11):1268–1274, 2008.

[71] Orli Yogev, Gilberto S. Almeida, Karen T. Barker, Sally L. George, Colin Kwok, James Campbell, Magdalena Zarowiecki, Dimitrios Kleftogiannis, Laura M. Smith, Albert Hallsworth, Philip Berry, Till Mocklinghoff, Hannah T.Webber, Laura S. Danielson, Bliss Buttery, Elizabeth A. Calton, Barbara M. da Costa, Evon Poon, Yann Jamina, Stefano Lise, Gareth J. Veal, Neil Sebire, Simon P. Robinson, John Anderson, and Louis Chesler. In vivo modeling of chemoresistant neuroblastoma provides new insights into chemorefractory disease and metastasis. *Cancer Research*, 79(20):5382–5393, 2019.

[72] Malcolm J. Moore. Clinical pharmacokinetics of cyclophosphamide. *Clinical Pharmacokinetics*, 20(3):194–208, 1991.

[73] Junwei Chen, Lijuan Ding, Wu Meng, Jinhua Yang, Chenglan Yan, Jianfang Xie, Luo Jing, Xiaofeng Li, and Zili Fu. Interspecies scaling and prediction of human clearance: Comparison of small- and macro-molecule drugs. *Xenobiotica*, 41(11):972–987, 2011.

[74] Xuan Wang, Haiyun Zhang, and Xiaozhuo Chen. Drug resistance and combating drug resistance in cancer. *Cancer drug resistance (Alhambra, Calif.)*, 2:141, 2019.

[75] Pedro M Enriquez-Navas, Jonathan W Wojtkowiak, and Robert A Gatenby. Application of evolutionary principles to cancer therapy. *Cancer research*, 75(22):4675–4680, 2015.

[76] Ariosto S Silva, Yoonseok Kam, Zayar P Khin, Susan E Minton, Robert J Gillies, and Robert A Gatenby. Evolutionary approaches to prolong progression-free survival in breast cancer. *Cancer research*, 72(24):6362–6370, 2012.

[77] Robert A Gatenby and Joel S Brown. Integrating evolutionary dynamics into cancer therapy. *Nature reviews Clinical oncology*, 17(11):675–686, 2020.

[78] Alberto Garaventa, Ulrike Poetschger, Dominique Valteau-Couanet, Roberto Luksch, Victoria Castel, Martin Elliott, Shifra Ash, Godfrey C. F. Chan, Geneviève Laureys, Maja Beck-Popovic, Kim Vettenranta, Walentyna Balwierz, Henrik Schroeder, Cormac Owens, Maja Cesen, Vassilios Papadakis, Toby Trahair, Gudrun Schleiermacher, Peter Ambros, Stefania Sorrentino, Andrew D. J. Pearson, and Ruth Lydia Ladenstein. Randomized trial of two induction therapy regimens for high-risk neuroblastoma: Hr-nbl1.5 international society of pediatric oncology european neuroblastoma group study, journal of clinical oncology. *Journal of Clinical Oncology*, 2021.

[79] Valeria Smith and Jennifer Foster. High-risk neuroblastoma treatment review. *Children*, 5(9):114, 2018.

[80] Vincristine dosing, indications, interactions, adverse effects, and more. https://reference.medscape.com/drug/oncovin-vincasar-pfs-vincristine-342097. Accessed: 2022-12-05.

[81] Cytoxan (cyclophosphamide) dosing, indications, interactions, adverse effects, and more. https://reference.medscape.com/drug/cytoxan-cyclophosphamide-342214. Accessed: 2022-12-05.

[82] A. K. Sarma. Introduction to genetic algorithm with a simple analogy. *Modeling and Optimization in Science and Technologies*, 2020.
